# Functional Characterization of Primordial Protein Repair Enzyme M38 Metallo-Peptidase From *Fervidobacterium islandicum* AW-1

**DOI:** 10.3389/fmolb.2020.600634

**Published:** 2020-12-17

**Authors:** Jae Won La, Immanuel Dhanasingh, Hyeonha Jang, Sung Haeng Lee, Dong-Woo Lee

**Affiliations:** ^1^Department of Biotechnology, Yonsei University, Seoul, South Korea; ^2^Department of Cellular and Molecular Medicine, Chosun University School of Medicine, Gwangju, South Korea; ^3^School of Applied Biosciences, Kyungpook National University, Daegu, South Korea

**Keywords:** M38 β-aspartyl peptidase, protein repair, starvation, type-I BAP, hyperthermophile stress responses, keratin degradation, *Fervidobacterium islandicum* AW-1

## Abstract

The NA23_RS08100 gene of *Fervidobacterium islandicum* AW-1 encodes a keratin-degrading β-aspartyl peptidase (*Fi*BAP) that is highly expressed under starvation conditions. Herein, we expressed the gene in *Escherichia coli*, purified the recombinant enzyme to homogeneity, and investigated its function. The 318 kDa recombinant *Fi*BAP enzyme exhibited maximal activity at 80°C and pH 7.0 in the presence of Zn^2+^. Size-exclusion chromatography revealed that the native enzyme is an octamer comprising a tetramer of dimers; this was further supported by determination of its crystal structure at 2.6 Å resolution. Consistently, the structure of *Fi*BAP revealed three additional salt bridges in each dimer, involving 12 ionic interactions that might contribute to its high thermostability. In addition, the co-crystal structure containing the substrate analog *N*-carbobenzoxy-β-Asp-Leu at 2.7 Å resolution revealed binuclear Zn^2+^-mediated substrate binding, suggesting that *Fi*BAP is a hyperthermophilic type-I IadA, in accordance with sequence-based phylogenetic analysis. Indeed, complementation of a Leu auxotrophic *E. coli* mutant strain (Δ*iadA* and Δ*leuB*) with *Fi*BAP enabled the mutant strain to grow on isoAsp-Leu peptides. Remarkably, LC-MS/MS analysis of soluble keratin hydrolysates revealed that *Fi*BAP not only cleaves the C-terminus of isoAsp residues but also has a relatively broad substrate specificity toward α-peptide bonds. Moreover, heat shock-induced protein aggregates retarded bacterial growth, but expression of BAP alleviated the growth defect by degrading damaged proteins. Taken together, these results suggest that the viability of hyperthermophiles under stressful conditions may rely on the activity of BAP within cellular protein repair systems.

## Introduction

Protein homeostasis (proteostasis), a balanced state between folded proteins and protein aggregates, is critical for cellular metabolism, physiology, and normal aging (Hutt and Balch, 2010). To maintain proteostasis, molecular chaperones control the folding of newly synthesized proteins, and the ubiquitin-proteasome system degrades misfolded and damaged polypeptides (Hipp et al., 2019). In eukaryotes, damaged proteins destined for degradation are ubiquitinated and subsequently degraded by the 26S proteasome (Goldberg, 2003). Like the ubiquitin-proteasome pathway in eukaryotes, prokaryotes also degrade abnormal proteins through a quality control network (QCN) consisting of chaperones and proteases (Goldberg, 1972; Mogk et al., 2011). To ensure protein homeostasis, bacteria should monitor the folding of proteins and prevent the accumulation of misfolded proteins via coordinated refolding or degradation (Mogk et al., 2011). Misfolded and abnormal proteins are produced for several reasons, including somatic mutations in genes that result in proteins failing to adopt the native folded structure, errors during transcription or translation, failure of the chaperone machinery, mistakes during the post-translational modification of proteins, and structural modifications caused by environmental changes (heat, oxidative agents, pH, or osmotic conditions), and induction of protein misfolding through seeding and cross-seeding mechanisms (Visick and Clarke, 1995; Moreno-Gonzalez and Soto, 2011). One of the major modifications of proteins is the deamination of asparaginyl or the isomerization of aspartyl residues through a cyclic intermediate, resulting in aspartyl or isoaspartyl (isoAsp) residues that contribute to inactivation, aggregation, and aging of proteins in tissue (Cournoyer et al., 2005). Indeed, isoAsp formation is associated with abnormal functioning of various proteins, including calmodulin, epidermal growth factor, and ribonuclease (Aswad, 1995). The formation of isoAsp can also decrease the biological activity of a protein drug, alter its susceptibility to proteolytic degradation, and induce autoimmunity (Aswad et al., 2000).

Bacteria possess enzymatic repair systems to prevent conformational damage to proteins resulting from proline isomerization, methionine oxidation, and formation of isoAsp residues (Visick and Clarke, 1995). Protein L-isoAsp *O*-methyltransferase (PIMT), one of the QCN proteins, is found in most archaea and Gram-negative eubacteria, as well as eukaryotes (Ryttersgaard et al., 2002). PIMT minimizes accumulation of atypical isoAsp residues by methylating the free α-carboxyl group of isoAsp residues (Reissner and Aswad, 2003). PIMT-deficient mice accumulate high levels of damaged proteins containing isoAsp residues, resulting in growth retardation and sudden early death (Kim et al., 1997). A similar PIMT protein repair enzyme was identified in the hyperthermophilic bacterium *Thermotoga maritima*, which grows optimally at 90°C. Accumulation of proteins with altered aspartyl and asparaginyl residues is believed to be detrimental to cell survival at elevated temperatures. The rate of isoAsp residue formation increases 910-fold at 90°C compared with 23°C (Ichikawa and Clarke, 1998); hence, modified or damaged protein (peptide) aggregates should be rapidly digested to prevent cellular damage and to provide a potential nutrient source to support cell survival in harsh environments. However, PIMT alone does not appear to be sufficient to prevent accumulation of protein aggregates due to its low conversion activity (Reissner and Aswad, 2003). Rather, isoAsp peptidase hydrolyzes the peptide bond directly to generate the β-carboxylate group of Asp (Marti-Arbona et al., 2005a), which is more efficient at handling peptides containing isoAsp residues.

The NA23_RS08100 gene encodes M38 β-aspartyl peptidase (BAP) in the feather-degrading bacterium *Fervidobacterium islandicum* AW-1 (Nam et al., 2002; Lee et al., 2015a). Among 57 genes encoding proteases in this organism, the NA23_RS08100 gene is most highly expressed when cells are grown under starvation conditions (Kang et al., 2020), suggesting that in addition to PIMT, the protein encoded by NA23_RS08100 might be involved in degrading isoAsp peptides under stressful conditions. To gain insight into the biological role of BAP as a QCN protein, we functionally and structurally characterized the protein product of the NA23_RS08100 gene in *F. islandicum* AW-1.

## Materials and Methods

### Phylogenetic Analysis

A phylogenetic tree was generated using the maximum likelihood method in MEGA X version 10.0.5, based on multiple sequence alignment of M38 β-aspartyl peptidase from *Fervidobacterium islandicum* AW-1 (*Fi*BAP) and homologs sharing ≥ 40% amino acid sequence identity. A bootstrap consensus tree was inferred from 1,000 replicates. Evolutionary distances were computed using the Poisson correction method and expressed as the number of amino acid substitutions per site. Amino acid sequence alignment was performed using ClustalX software V2.1. Sequence similarities and secondary structure information from aligned sequences were generated using ESPript 3.0 (Robert and Gouet, 2014).

### Cloning and Expression of the NA23_RS08100 Gene

Genomic DNA was isolated from *F. islandicum* AW-1, purified using a genomic DNA extraction kit according to the manufacturer's instructions (Qiagen, Hilden, Germany), and then used as template for PCR amplification. The NA23_RS08100 gene encoding *Fi*BAP was amplified by PCR using forward (5′-GCTAGCATGATAAAAATTATAAAGAACG-3′) and reverse (5′-CTCGAGTCAAAATTCAAAGTTTAAGTTC-3′) primers (underlined sequences represent the restriction sites for *NheI* and *XhoI*). The PCR product was digested with *Nhe*I and *Xho*I and cloned into the pET-28a(+) vector (Novagen, San Diego, CA), yielding pET-*Fi*BAP. The resulting plasmid encodes the target gene fused to a 6 × His-tag at the N-terminus. For expression of recombinant *Fi*BAP, *E. coli* BL21 (DE3) cells transformed with pET-*Fi*BAP were grown at 37°C in 1 L of Luria-Bertani (LB) medium containing kanamycin (50 μg/mL) to an optical density at 600 nm (OD_600_) of 0.5–0.8. After induction with 1 mM Isopropyl-1-thio-β-D-galactopyranoside (IPTG), cells were grown overnight at 37°C, then harvested by centrifugation (10,000 × g, 20 min, 4°C) and stored at −80°C.

### Purification of *Fi*BAP

The harvested cells were resuspended in 20 mM Tris-HCl buffer containing 500 mM NaCl, 5 mM imidazole, and 1 mM phenylmethylsulfonyl fluoride (PMSF) (pH 7.9) and disrupted by sonication. After centrifugation at 10,000 × g for 20 min, the supernatant was heated at 60°C for 30 min then centrifuged at 10,000 × g for 20 min to remove denatured *E. coli* proteins. The supernatant was applied to Ni^2+^-affinity resin (10 mL) equilibrated with the same buffer, and His-tagged protein was eluted with 20 mM Tris-HCl buffer containing 500 mM NaCl and 250 mM imidazole. Samples were loaded on a Superdex 200 10/300 GL column (GE Healthcare, USA) equilibrated with 25 mM Tris-HCl buffer (pH 7.5) containing 150 mM NaCl (Supplementary Table 1). The major fractions were analyzed by 12% sodium dodecyl sulfate polyacrylamide gel electrophoresis (SDS-PAGE) and visualized using Coomassie Blue staining.

To monitor the recombinant *Fi*BAP, western blotting was performed using a 6 × His tag monoclonal antibody (ThermoFisher Scientific, USA). Each sample was subjected to 12% SDS-PAGE, electrophoretically transferred to a polyvinylidene fluoride (PVDF) membrane (Bio-Rad, Hercules, CA, USA), blocked with 5% skim milk in TBST buffer (20 mM Tris-HCl, pH 7.5, 150 mM NaCl, and 0.05% Tween 20) at 4°C overnight, and incubated with horseradish peroxidase-conjugated 6 × His epitope monoclonal antibody (1:1,000 dilution) for 2 h at room temperature. The membranes were then washed three times with TBST buffer for 10 min and were developed using a WESTSAVE Up western blotting detection system (AbFrontier, Seoul, Korea).

### Preparation of Recombinant Feather Keratins Expressed in *E. coli* Cells

Recombinant feather keratins were prepared as described previously (Jin et al., 2017) and used as native substrates. Briefly, *E. coli* cells expressing recombinant feather keratins were resuspended in lysis buffer (50 mM NaH_2_PO_4_, 300 mM NaCl, 10 mM imidazole, 1 mM PMSF, pH 8.0) and disrupted by sonication. After centrifugation at 10,000 × g for 30 min, expressed keratins in the form of inclusion bodies were resuspended in lysis buffer containing 8 M urea and 1 mM PMSF, incubated on ice for 1 h, and centrifuged at 16,000 × g for 30 min. Supernatants were filtered through a 0.45 μm membrane, then applied to a 10 mL Ni-NTA agarose resin column (Qiagen, Germany) equilibrated with lysis buffer containing 8 M urea. Fractions containing unfolded keratins were eluted with 250 mM imidazole, concentrated using an Amicon Ultra-3K device (Millipore, USA), and buffer-exchanged by step-wise dialysis against 50 mM Tris-HCl (pH 8.0) at 4°C. Dialyzed samples were centrifuged at 10,000 × g for 30 min to remove insoluble material, and the resulting supernatants containing refolded keratins were concentrated using an Amicon Ultra-3K device (Millipore).

### Enzyme Activity Assay

*Fi*BAP activity was determined by measuring the increase in free amino acids (Rosen, 1957). Reaction mixtures (0.16 mL) containing 50 mM 4-(2-hydroxyethyl)-1-piperazineethanesulfonic acid (HEPES) buffer (pH 8.0), 500 ng/mL enzyme, and 1 mM β-Asp-Leu (β-DL) as a synthetic substrate were incubated at 80°C for 15 min. The reaction was stopped by adding trichloroacetic acid (TCA) reagent. After an additional 15 min incubation at 100°C for color development, followed by cooling on ice, the absorbance was measured at 570 nm. One unit of *Fi*BAP activity was defined as the amount of enzyme that produced 1 μmol of product per min under the assay conditions (U/mg). Additionally, to assess the proteolytic activity of the purified enzyme, we measured the increase in free amino acids using the ninhydrin assay (Rosen, 1957) with casein and gelatin as substrates.

### Biochemical Characterization of *Fi*BAP

The temperature dependence of *Fi*BAP activity was measured using the standard protocol but the reaction temperatures was varied from 40 to 98°C. To determine the effect of pH on *Fi*BAP activity, reaction mixtures (0.16 mL) were incubated at 80°C under standard assay conditions but HEPES buffer was replaced by 50 mM sodium acetate buffer (pH 4.0–6.0), HEPES buffer (pH 6.0–8.0), borate buffer (pH 8.0–10.0), or sodium bicarbonate buffer (pH 9.0–10.0). All pH values were adjusted at room temperature, and ΔpK_a_/ΔTs (in which the latter term is the change in temperature) for each buffer was taken into account when the results were analyzed.

To determine the kinetic parameters, assays were performed in 50 mM HEPES (pH 8.0) containing 1 mM CoCl_2_, 500 ng/mL enzyme, and 1–32 mM β-DL substrate. Reaction mixtures were incubated for 10 min at 80°C and stopped by cooling on ice. Kinetic parameters were obtained by fitting the empirical data to the Michaelis-Menten equation using Origin 8.0 software.

To analyze the effect of metals, purified *Fi*BAP was treated with 20 mM EDTA at room temperature for 2 h to remove metal ions, followed by overnight dialysis against 20 mM Tris-HCl (pH 7.5) at 4°C with three changes of buffer. The divalent metal ion content of both as-isolated and EDTA-treated samples were determined by high-resolution inductively-coupled plasma mass spectrometry (ICP-MS) on a PlasmaQuad 3 instrument. To assess the effects of various metal ions on *Fi*BAP activity, the EDTA-treated samples were preincubated with 1 mM CoCl_2_·6H_2_O, MnCl_2_·4H_2_O, MgCl_2_·6H_2_O, CaCl_2_·2H_2_O, ZnCl_2_·6H_2_O, CuCl_2_·2H_2_O, FeCl_2_·6H_2_O, or NiCl_2_·6H_2_O for 15 min at 50°C. The residual activity was measured in triplicate under standard assay conditions.

### Crystallization and Structure Determination of *Fi*BAP

Preliminary crystallization trials were performed using purified *Fi*BAP (10 mg/mL) in an appropriate buffer (20 mM Tris-HCl pH 7.4, 50 mM NaCl) mixed with protein crystallization solutions (Hampton Research and Wizard; 1:1 volume ratio) by the hanging drop vapor-diffusion method at 20°C (293 K) as described previously (Lee et al., 2015b). The initial *Fi*BAP crystals were further improved by modifying the Crystal Screen Lite 34 conditions (0.1 M sodium acetate pH 4.6 and 1 M sodium formate). *Fi*BAP was co-crystallized with substrate analog *N*-carbobenzoxy-β-Asp-Leu (Cbz-β-DL) at a molar ratio of 1:1.2, and crystals of the complex were obtained in modified PEG/Ion screen 2–32 conditions (2% v/v tacsimate pH 5.0, 0.1 M sodium citrate tribasic dihydrate, pH 5.6; 16% w/v polyethylene glycol 3350). Crystals were soaked for 30 s in a cryosolution containing mother liquor plus 20% glycerol, then frozen in liquid nitrogen prior to synchrotron radiation diffraction experiments.

X-ray diffraction data were collected from *Fi*BAP crystals on beamline 7A at the Pohang Light source (Pohang, Korea) using an ADSC Q270 detector, with an oscillation of 1.0° and a 1 s exposure per frame over a 360° range at a wavelength of 0.97934 Å. Crystals of *Fi*BAP with and without β-DL as substrate grown using the above conditions diffracted to 2.6 Å and 2.7 Å, respectively. Diffraction data were processed using the HKL-2000 program (HKL Research Inc.). In the case of the ligand-free structure, molecular replacement (MR) was performed with *Ec*IadA (PDB: 1ONW) as the search model using the CCP4 program MOLREP (Vagin and Teplyakov, 2010). The initial model was further refined using the PHENIX (Afonine et al., 2010) and REFMAC5 (Murshudov et al., 2011) programs to achieve a model with R_work_ and R_free_ values of 16.9 and 22.1%, respectively. In the case of the Cbz-β-DL-bound *Fi*BAP diffraction dataset, to enhance the overall data quality, two native datasets of equivalent diffraction limits were combined using the Scale_and_Merge function of the PHENIX suite (Adams et al., 2010). MR was performed for the Cbz-β-DL-bound *Fi*BAP structure with the refined crystal structure of ligand-free *Fi*BAP as the search model using the Phaser program (McCoy, 2007). The chemical coordination file for β-DL was built using Coot (Emsley and Cowtan, 2004) and eLBOW (Moriarty et al., 2009). The ligand was fitted into the map, and several rounds of refinement were performed using the REFMAC5 and PHENIX programs, yielding R_work_ and R_free_ values of 19.7 and 27.5%, respectively. The structures of *Fi*BAP with and without Cbz-β-DL have been deposited in the Protein Data Bank (PDB) under accession codes 7CDH and 7CF6, respectively.

### Construction of Leucine Auxotrophic *E. coli* Mutants

The leucine (Leu) auxotrophic *E. coli* BL21 (DE3) strain was constructed by deleting the *leuB* gene encoding 3-isopropyl malate dehydrogenase, which is involved in leucine biosynthesis, by the RED/ET recombination method using a Quick & Easy *E. coli* Gene deletion kit (Gene Bridges) according to the manufacturer's instructions. A functional cassette flanked with FRT and homology arms was generated by PCR using the FRTPGK-gb2-neo-FRT fragment as a template and primers 5′-AGTTGCAACGCAAAGCTCAACACAACGAAAACAACA-AGGAAACCGTGTGAAATTAACCCTCACTAAAGGGCGG-3′ (forward) and 5′-GTACACAACGTGAGCGTCGAACAAT-TTTTCGTATAACGTCTTAGCCATGATAATACGACTCACT-ATAGGGCTCG-3′(reverse). The kanamycin selection marker was removed by transforming cells with FLP recombinase expression plasmid 706-FLP (Gene Bridges). After selecting mutants lacking the selection marker cassette by streaking each colony on LB agar plates with and without 50 μg/mL kanamycin, deletion of the *leuB* gene was confirmed by PCR with primers 5′-GCTAACTACAACGGTCGCCGCTTCCACGGCGTC-3′ (forward) and 5′-GGCGCGCAGACCATCGAACGCCTGCGG-TGAGG-3′ (reverse).

For functional complementation of *Fi*BAP, a functional cassette flanked with FRT and homology arms was generated to delete the *iadA* gene encoding *Ec*IadA using primers 5′-CATTGCTGTCGATCTGGGTTATGCAGCTTATTGTTTAA-CAAGGAGTTACCAATTAACCCTCACTAAAGGGCGG-3′(forward) and 5′-TCAGCCGCCCTTGCGGGCATTCTACG-TCCATTCGGGCGGCTGACAACCGTTAATACGACTCACT-ATAGGGCTCG-3' (reverse). In addition, primers 5′-GTC-GGTCGCTGCCTGGGGACAGCCGAAGTG-3′ (forward) and 5′-CTGAGAGTTGCAGCGGCGTTACCTGGCGG-3′ (reverse) were used to confirm the *iadA* deletion mutants.

### Complementation of *iadA* Deletion by *Fi*BAP

To monitor the growth phenotypes of the Leu auxotrophic *E. coli* strains harboring the pET28a-*Fi*BAP and the pET28a-*iadA* plasmids, each protein-overexpressing strain was streaked on appropriate minimal M9 agar plates containing 50 μg/mL kanamycin and 1 mM IPTG supplemented with different compositions of amino acids (19 amino acids with and without Leu) in the presence and absence of 1.6 mM β-DL. To investigate the effect of heat shock on bacterial growth retardation, each strain was grown at 37°C to an OD_600_ of 1.2–1.5 in LB medium containing kanamycin (50 μg/mL), and then incubated at 44°C for 0.5 h or longer until cells entered the stationary phase.

To determine the total protein content of soluble (supernatant) and insoluble (pellet) fractions, cells were harvested before (mid-exponential phase) and after (stationary phase) heat treatment. The same amount of cells, as judged by the OD_600_ value, was resuspended in 50 mM Tris-HCl buffer (pH 8.0) and disrupted by sonication. After centrifugation at 10,000 × g for 30 min, the protein concentration of fractionated samples was determined using the bicinchoninic acid (BCA) assay (Smith et al., 1985), and each subcellular fractions was visualized on the 12% acrylamide gel for SDS-PAGE analysis.

### LC-MS/MS Analysis

Keratin hydrolysates were analyzed by reversed-phase HPLC-ESI-MS/MS using a NanoLC-2D Ultra system (Eksigent, Dublin, CA, USA) coupled to a LTQ-XL mass spectrometer (Thermo Fisher Scientific, Bremen, Germany) in direct injection mode. Briefly, after a 5 μL injection, keratin hydrolysates were loaded onto a reversed-phase ProteoPepII C18 column (5 μm, 300 Å pore size, 0.15 × 25 mm; New Objective IntegraFrit, Scientific Instrument Services, Inc., Ringoes, NJ, USA) and eluted onto a Molex Polymicro Flexible Fused Silica Capillary Tubing (I.D. 75 μm, O.D. 375 μm) at a flow rate of 0.4 μL/min. The gradient consisted of mobile phase A [0.1% formic acid (v/v) in water], mobile phase B [0.1% formic acid (v/v) in acetonitrile], 2% solvent B (0–1 min), 5% solvent B (1–62 min), 35% solvent B (62–65 min), 60% solvent B (65–70 min), and 2% solvent B in A (70–90 min) with a total runtime of 90 min, including mobile phase equilibration.

MS analysis of peptide eluents was performed on a LTQ-XL system (ThermoFisher Scientific) in positive-ion mode with a nano ion spray voltage of 2.3 kV, a scan range of 300–1,800 (m/z), a curtain gas pressure of 20 psi, a nebulizer gas pressure of 6 psi, and an interface heater temperature of 200°C. Full-scan MS spectra were acquired from 35 precursors selected for MS/MS analysis from the 300 to 1,800 m/z range, utilizing a dynamic exclusion of 30 s. The IDA collision energy (CE) parameter script, selecting up to 35 precursors with charge states of +2 to +3, was employed to automatically control the CE.

Data were processed using MSConvert (ProteoWizard). This software converts raw data (.RAW format) into peak lists (.mgf format). The FASTA database employed contained the *Gallus gallus* keratin sequence, and this afforded the opportunity to employ the target decoy database search strategy (Elias and Gygi, 2007). Data containing both MS and MS/MS information were uploaded into Xcalibar software (ThermoFisher Scientific) and used to generate MS-extracted ion chromatograms (XICs) for each identified peptide. For the MS-GF search, carbamidomethyl (C) was set as a fixed modification and oxidation (M) was set as a variable modification. The peptide tolerance was ±4.0 Da, and the MS/MS tolerance was ±1 Da. The software algorithm simultaneously searched all modifications listed in UniMod (http://www.unimod.org/) (Shilov et al., 2007). False discovery rate (FDR) analysis was also performed using integrated tools in PeptideShaker (CompOmics), which generated.mgf files that were subsequently searched against the current *G*. *gallus* keratins SwissProt database using MS-GF.

### Differential Scanning Calorimetry (DSC) Measurement

Calorimetric measurements were performed using a VP-DSC microcalorimeter (Microcal Inc., GE Malvern, Worcestershire, UK). All scans were run at pH 7.4 in 10 mM potassium phosphate buffer containing 150 mM NaCl, over a temperature range from 10 to 130°C at a rate of 90°C/h. The cell volume was 0.8 mL. Potassium phosphate buffer was used for baseline scans, and apparent melting temperature (*T*_m_) values of *Fi*BAP (25 μM) were determined.

### Analysis of Isoaspartic Acid Residues

An ISOQUANT Isoaspartate Detection Kit (Promega) was used for the detection of isoaspartate (isoAsp) according to the manufacturer's instructions. Each cell lysate (2 μg) from heat-shocked strains was incubated at 30°C for 30 min in the presence of L-isoaspartyl (isoAsp) methyltransferase (PIMT) and S-adenosyl methionine (SAM) in the reaction buffer, and the stop solution was then added to halt the reaction. The reaction mixture (40 μl) was loaded onto a reversed-phase YMC-Triart C18 column (250 × 4.6 mm I.D. S-5 μm, 12 nm) equilibrated with 90% 50 mM potassium phosphate (pH 6.2) and 10% methanol. The amount of isoAsp was determined by quantifying eluted S-adenosyl homocysteine (SAH).

## Results

### *In silico*-Based Annotation of the NA23_RS8100 Gene Product

**Figure 1:**
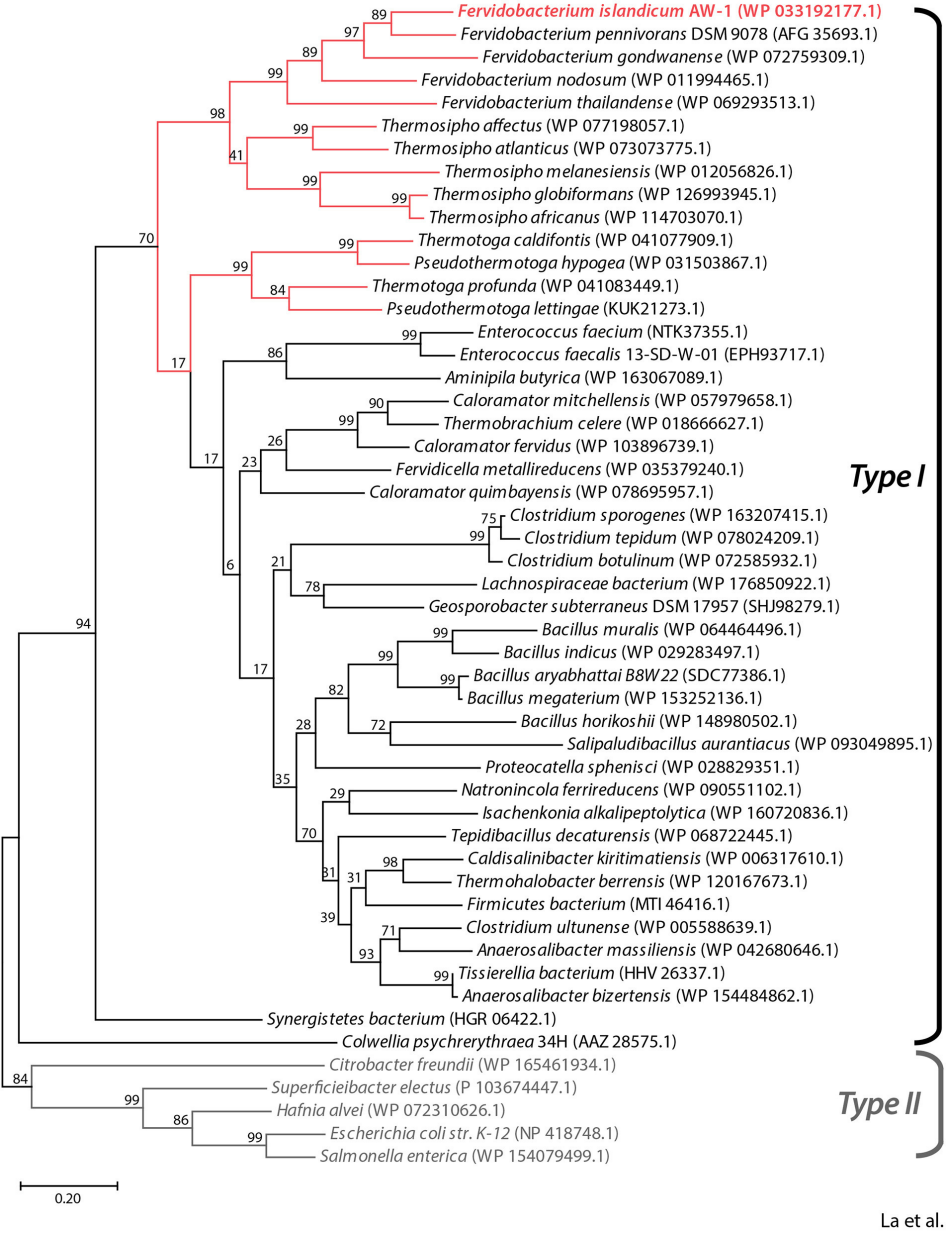
Phylogenetic analysis of *Fi*BAP and its homologs. The phylogenetic tree was constructed based on protein sequence alignments of *Fi*BAP and 50 BAP homologs using the maximum likelihood method in MEGA X. Bootstrap values are indicated at the branch points. Red lines indicate BAP derived from hyperthermophilic bacteria. The scale bar indicates a branch length equivalent to 0.2 changes per amino acid. NCBI protein database accession numbers for each individual protein sequence are indicated in parentheses.

To predict the functional role of the NA23_RS8100 gene product, we performed a BLASTP search using the deduced amino acid sequence to identify homologs. Multiple sequence alignment of the NA23_RS8100 gene product with β-aspartyl peptidases (BAPs) revealed that its amino acid sequence shares high levels of sequence identity with BAP from *Fervidobacterium* species (≥65%) and other genera including *Thermosipho* (≥55%), *Pseudothermotoga* (≥51%), *Thermotoga* (≥51%), *Clostridium* (≥49%), *Bacillus* (≥47%), and *Enterococcus* (≥49%; Figure 1 and Supplementary Figure 1). In addition, the deduced amino acid sequence of the NA23_RS08100 gene product shares pronounced sequence identity with mesophilic BAPs from *Escherichia coli* (*Ec*IadA; 39% sequence identity; PDB:1ONW) and *Colwellia pshchrerythraea* 34H (*Cp*IadA; 41% sequence identity; PDB:5XGW), for which crystal structures are available. Remarkably, all BAP homologs derived from extremophiles belong to the Type I isoaspartyl dipeptidase (IadA) family, members of which possess a conserved Glu residue for metal binding in the active site (Supplementary Figure 1). Moreover, phylogenetic analysis showed that BAP from *F. islandicum* AW-1 (*Fi*BAP) has a highly close evolutionary relationship with homologs from extremophilic *Fervidobacterium* and *Thermosipho*, whereas extremophilic *Fi*BAP has diverged away from the Type II IadAs of mesophiles in the order *Enterobacteriales* and further away from *Ec*IadA and *Cp*IadA members of the BAP family (Figure 1). These results led us to tentatively conclude that the protein encoded by the NA23_RS8100 gene in *F. islandicum* AW-1 is β-aspartyl peptidase (subsequently named *Fi*BAP), representing an ancient form of IadA that has diverged from Type II IadA.

### Biochemical Characterization of *Fi*BAP

**Figure 2:**
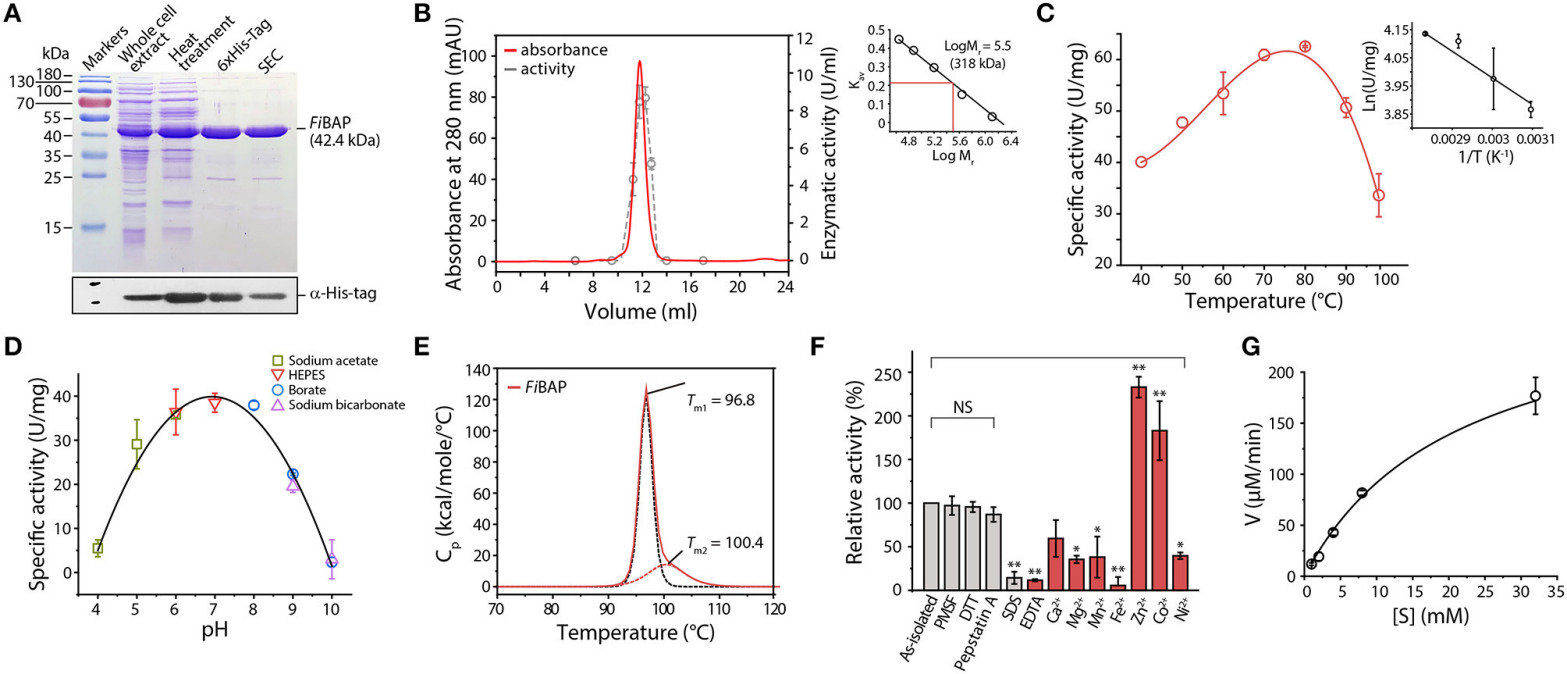
Biochemical properties of *Fi*BAP. **(A)** Purification of recombinant *Fi*BAP analyzed by 12% SDS-PAGE. **(B)** Size-exclusion chromatography of *Fi*BAP using a Superdex 200 pg 10/300 column and the enzyme activity of the eluents. The column was calibrated with blue dextran (2,000 kDa), thyroglobulin (669 kDa), ferritin (440 kDa), aldolase (158 kDa), conalbumin (75 kDa), and ovalbumin (44 kDa) as standards (Supplementary Figure 2). The calibration curve shows Log *M*_r_ and K_av_ values for each standard protein. **(C)** Temperature dependence of *Fi*BAP activity. **(D)** Dependence of *Fi*BAP activity on pH. **(E)** DSC analysis of *Fi*BAP. **(F)** Effects of divalent metal ions and inhibitors on *Fi*BAP activity. Group differences were assessed using one-way analysis of variance (ANOVA), followed by Tukey HSD test (^*^p < 0.05 and ^**^p < 0.01 vs. as-isolated). **(G)** Kinetic analysis of *Fi*BAP. The kinetic parameters for purified *Fi*BAP were estimated by steady-state kinetics analysis using β-DL as substrate.

We successfully expressed the NA23_RS08100 gene as a C-terminal hexahistidine (6 × His)-tagged fusion protein in *E. coli* BL21 (DE3) cells. To facilitate subsequent purification, *E. coli* lysates were heat-treated to remove most of the endogenous proteins, and the resulting supernatants containing soluble *Fi*BAP were applied to a Ni^2+^-affinity column. The purified 6 × His-tagged *Fi*BAP fusion protein yields an intact recombinant protein with an estimated *M*_r_ of 42,000 by SDS-PAGE (Figure 2A). However, size exclusion chromatography using a Superdex 200 column suggested that the native form of *Fi*BAP is as an octameric protein with an apparent *M*_r_ of 318,000 (Figure 2B).

Unlike typical endo-proteases, *Fi*BAP displayed minimal activity toward casein and gelatin as substrates, but it was active against β-Asp-Leu (β-DL), with an apparent optimal temperature of 80°C (Figure 2C) and an apparent optimum pH of 7.0 at 80°C (Figure 2D). To further assess the structural stability of *Fi*BAP, we used DSC to determine the T_m_, revealing a major midpoint of the thermal transition (T_m_ = 96.8 ± 0.5°C) and a minor one at 100.4 ± 1.44°C (Figure 2E).

Since *Ec*IadA is a metalloprotease, we performed ICP-MS analysis of as-isolated *Fi*BAP, demonstrating that it contained Zn^2+^ (1.14 ± 0.37 mol of metal per mol of monomer). To investigate the effects of inhibitors on *Fi*BAP activity, as-isolated enzyme was pre-incubated with various inhibitors and its residual activity was measured. Dithiothreitol, PMSF, and Pepstatin A had little inhibitory effect on *Fi*BAP, but 1% SDS and 1 mM EDTA inhibited enzyme activity (Figure 2F). Subsequently, after a 15 min preincubation of EDTA-treated and dialyzed *Fi*BAP with various metal ions at 50°C, the residual activity was measured under standard assay conditions, demonstrating that *Fi*BAP activity was significantly increased in the presence of Zn^2+^ and Co^2+^ compared with that in the absence of divalent metal ions, suggesting that this enzyme may be a metalloenzyme.

Additionally, we performed steady-state kinetics analysis of *Fi*BAP with β-DL as substrate, yielding *K*_m_ and V_max_ values of 5.23 ± 1.59 mg/mL and 286.33 ± 65.7 μM/min, respectively (Figure 2G).

**Figure 3:**
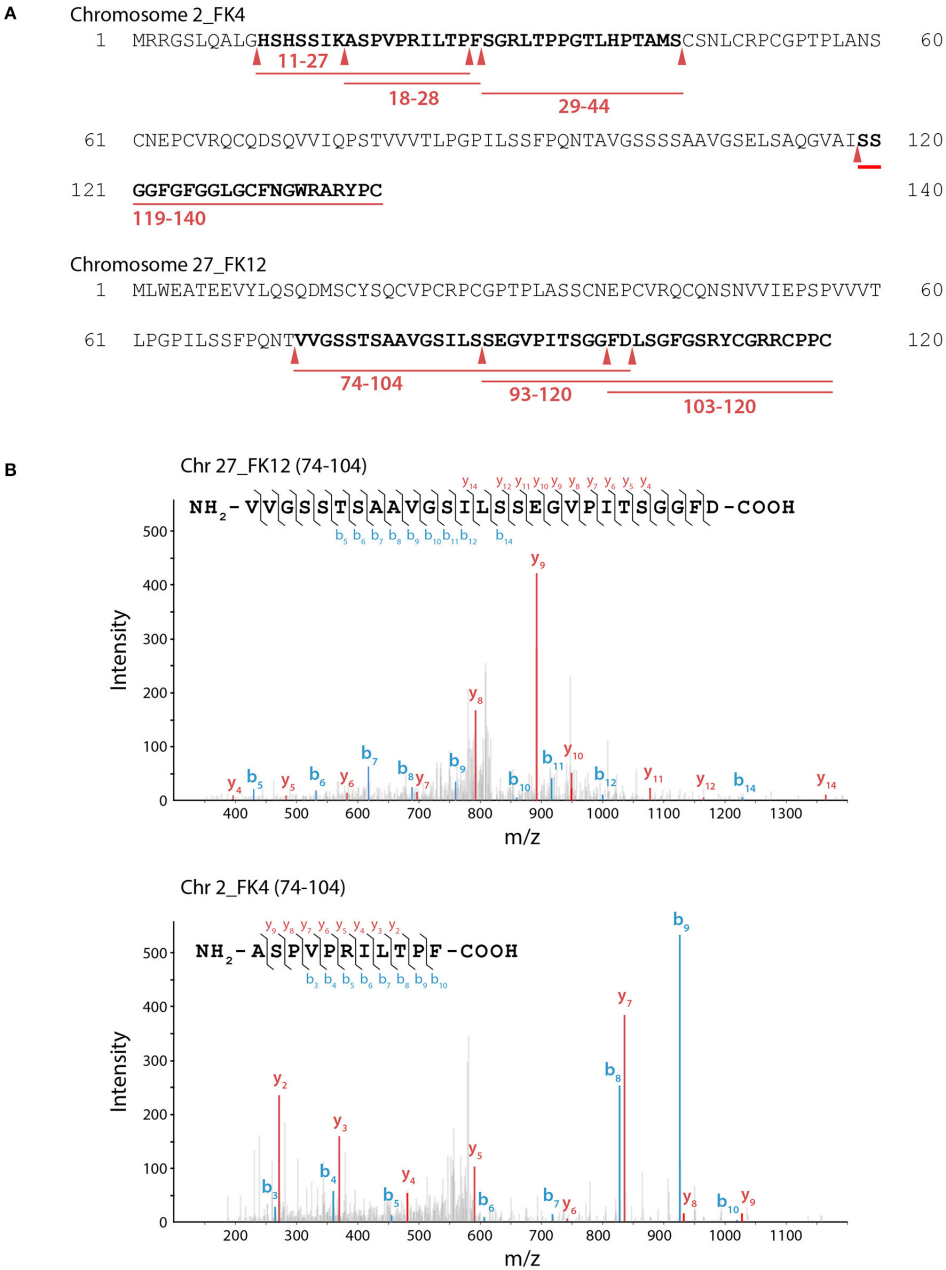
LC-MS/MS analysis of keratin hydrolysates generated by *Fi*BAP. **(A)** Keratinolytic peptides matched with soluble Chr2_FK4 and Chr27_FK12 β-keratins are underlined in red, and their coverage sequences are depicted in bold. Red arrows indicate the putative cleavage site of *Fi*BAP from the identified peptide sequences. Purified recombinant feather keratin and *Fi*BAP were incubated for 18 h at 80°C in 50 mM Tris-HCl (pH 7.5). The enzyme/substrate ratio was 1:50 (w/w). **(B)** Representative MS spectra of keratinolytic peptides and fragment ions of detected peptides from recombinant feather keratins. The identified fragment ions from peptide sequences are labeled in the spectra and indicated on the peptide sequence above. The b (blue) and y (red) peaks correspond to the majority of the N- and C- terminal ions resulting from fragmentation of each peptide, respectively.

Notably, *Fi*BAP is the most abundant and highly expressed enzyme when *F. islandicum* AW-1 is grown on feathers under starvation conditions (Kang et al., 2020). Although a MEROPS (http://merops.sanger.ac.uk) database search provided some information on hierarchical, structure-based classification of the peptidases, it did not yield the predicted cleavage pattern for M38 BAP. To further investigate the hydrolysates resulting from *Fi*BAP degradation, we performed LC-MS/MS analysis using soluble keratins as native substrates (Supplementary Table 2). Remarkably, all peptides hydrolyzed by *Fi*BAP matched the keratin sequences derived from *G. gallus* sequences, indicating that *Fi*BAP can cleave not only the peptide bond between Asp and Leu, but also other α-peptide bonds between hydrophobic, aromatic, and hydrophilic amino acid residues at the C-terminus (Figure 3), as were the cases of the *Ec*IadA and BAP from rat tissue (Dorer et al., 1968; Gary and Clarke, 1995). This result suggests that *Fi*BAP is a beta-aspartyl peptidase with a relatively broad substrate specificity toward α-peptide bonds.

### Overall Architecture of *Fi*BAP

**Figure 4:**
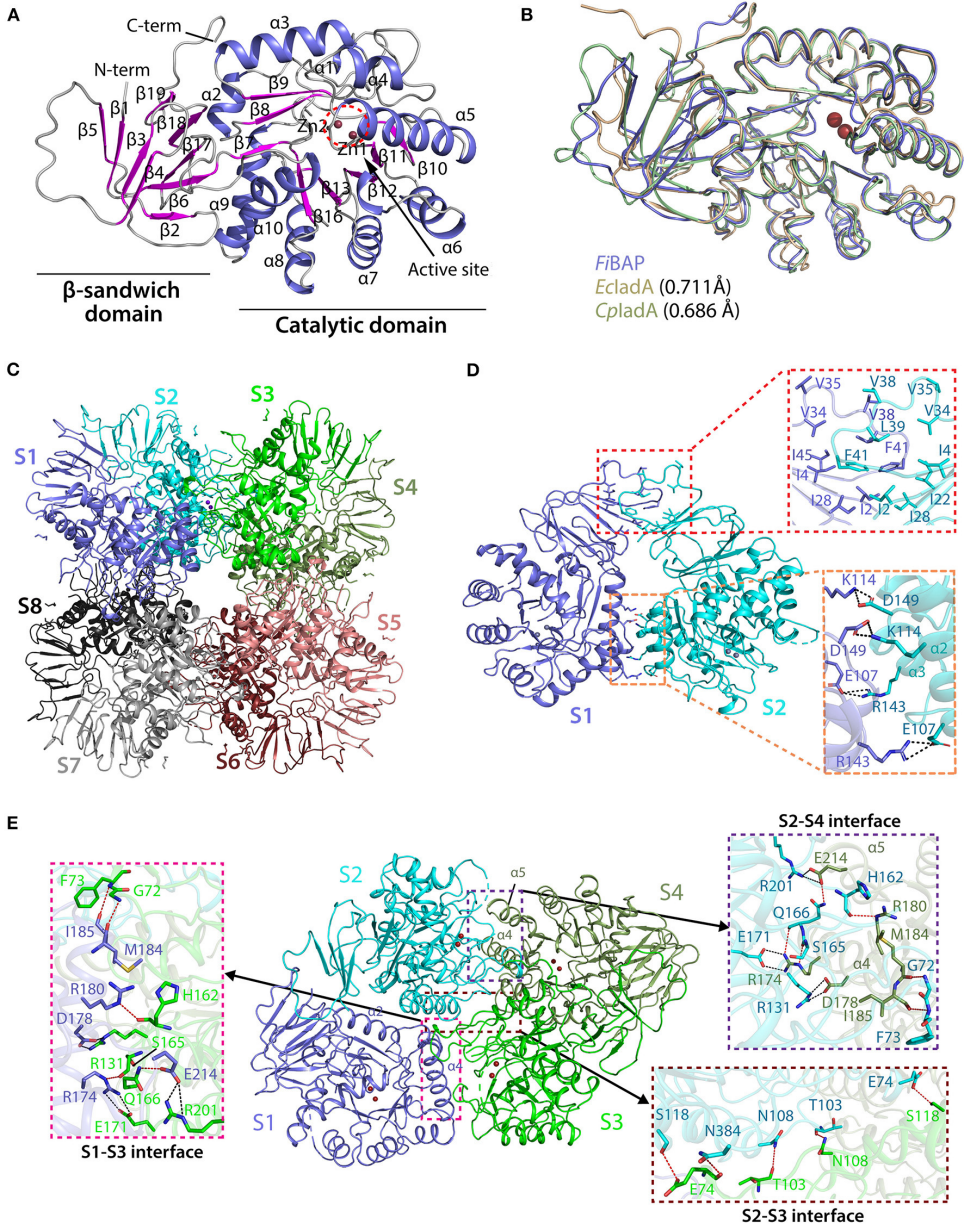
Crystal structure of *Fi*BAP. **(A)** Domain structure of monomeric *Fi*BAP. The monomeric subunit of *Fi*BAP consists of two domains; an N-terminal β-sandwich domain and a C-terminal catalytic domain. The structural elements from the N- to C- termini of the protein are labeled. The active site (marked as a dotted red circle) contains two zinc metals (Zn1 and Zn2) located in the catalytic domain. **(B)** Structural alignments of *Fi*BAP with structural homologs *Ec*IadA (PDB ID 1ONW) and *Cp*IadA (5XGW). RMSD values are indicated in parentheses. **(C)** Quaternary structure of *Fi*BAP comprising dimers of two tetramers. Symmetry-related molecule analysis reveals *Fi*BAP to be an octamer of eight subunits (S1–S8, subunits colored differently). **(D)** Interactions at the interface between each subunit in a dimer. Dimers formed from two subunits (S1 and S2) are shown. The top red box shows hydrophobic interactions between β-sandwich domains, and the bottom salmon box shows salt bridges formed between catalytic domains of each subunit. **(E)** Dimer-dimer interactions. Interface interactions between the S1–S2 dimer (slate blue and cyan) and the S3–S4 dimer (green and smudge) are shown. The molecular interactions at three contact points, the S2–S4 interface (purple box), the S2–S3 interface (brown box), and the S1–S3 interface (magenta box), are shown in zoomed representation.

Crystal structures of the ligand-free form of *Fi*BAP and the ligand-bound form bound with *N*-carbobenzoxy-β-Asp-Leu (Cbz-β-DL) as a substrate analog were determined at resolutions of 2.6 and 2.7 Å, respectively (Table 1). Cbz-β-DL-free and -bound structures belonged to I422 and P22_1_2 space groups, with one and four subunits in the asymmetric unit, respectively. The overall structure of each monomer can be divided into two distinct domains; an N-terminal β-sandwich domain (M1–G56; G344–E386) and a C-terminal catalytic domain (L57–K343), that are mainly involved in dimerization and catalysis, respectively (Figure 4A). The β-sandwich domain located at the N-terminus of the molecule mainly comprises nine β-sheets (β1–β6, β17–β19) arranged in two layers with β1, β3, β4, and β5 in one layer and β2, β6, and β17–β19 in the other layer (Figure 4A). Inter-layer interactions may stabilize the β-sandwich domain consisting of loops and β-strands through hydrophobic interactions and a salt bridge between K376 in β19 and E44 in β5. The catalytic domain at the C-terminus of the molecule folds into a (β/α)_8_ triosephosphate isomerase (TIM)-barrel motif. Specifically, eight β-sheets (β7–β13) surrounded by nine α-helices (α2–α10) form the central core containing the substrate-binding site harboring two Zn^2+^ ions (Figure 4A).

**Table 1:** Crystallographic data collection statistics for *Fi*BAP.

	***Fi*BAP (ligand-free)**	***Fi*BAP (ligand-bound)**
**PDB ID**	**7CDH**	**7CF6**
**Diffraction data statistics**		
Beamline	PLS-7A	PLS-11C
Wavelength (Å)	0.97934	0.98461
Temperature (K)	100	100
Space group	I422	P2 2_1_ 2
**Cell parameters**		
*a, b, c* (Å)	143.9, 143.9, 119.6	78.6, 150.4, 151.9
*α, β, γ* (°)	90.0, 90.0, 90.0	90.0, 90.0, 90.0
Data resolution (Å)	30.0–2.6 (2.69–2.6)	47.6–2.7 (2.75–2.7)
Completeness (%)	97.7 (100.0)	99.7 (99.4)
Redundancy	22.9 (24.6)	10 (6.9)
Total reflections	439,332	466,992
Unique reflections	18,969 (1908)	36,967 (1041)
${\text{R}}_{\text{merge}}^{\text{a}}$	7.3 (82.4)	7.4 (155.2)
${\text{CC}}_{1/2}^{\text{b}}$	0.997 (0.963)	0.997 (0.368)
Matthew's coefficient (Å^3^ Da^−1^)	3.66	2.67
Solvent content (%)	66.37	54.04
Average I/Sigma (I)	50.3 (5.54)	9.0 (1.0)
No. of chains per asymmetric unit	1	4
**Refinement**		
R_work_ (%)	19.9 (27.9)	19.7 (34.5)
R_free_ (%)	24.4 (36.4)	27.5 (37.7)
Protein residues/Ligands	360/0	1497/4
**RMSD**		
Angles (°)	1.35	1.97
Lengths (Å)	0.011	0.015
Average B-factors (Å^2^)	40.30	49.98
**Ramachandran plot**		
Most favored regions (%)	96	92.74
Allowed regions (%)	3.43	6.25
Outliers (%)	0.57	1.02

*Values in parentheses correspond to the highest resolution shell*.

a *R_merge_ = Σ_hkl_ Σ_i_ / I_i_(hkl) – < I(hkl)>| / Σ_hkl_ Σ_i_ I_i_(hkl), where I_i_(hkl) and < I(hkl)> are the intensity of an individual reflection and the mean value of all measurements of an individual reflection, respectively*.

b *CC_1/2_ values are the correlations between intensities from random half-data sets*.

A structural similarity search using the DALI server revealed that *Fi*BAP is very closely related to *Ec*IadA, a Type II IadA enzyme that has evolved in the direction of allowing post-translational modification (PTM) of the active site for metal binding and catalytic activity (Park et al., 2017). Structural neighbors identified in the *Fi*BAP query structure include IsoAsp dipeptidase, dihydropyrimidinase-related protein, enamidase, allantoinase, and dihydropyrimidinase, all of which are hydrolases. The structural architecture of *Fi*BAP was compared with previously determined structures of homologs (*Ec*IadA and *Cp*IadA). All monomers were superimposed with an average root mean square deviation (RMSD) of 0.705 Å for all Cα atoms (Figure 4B), suggesting that the overall scaffold of *Fi*BAP is similar to that of its homologs. Among these BAPs, structural elements are highly conserved, except for a minor difference in the flexible loop connecting β4 and β5 (Figures 4A,B), which might be of no discernable biological significance.

Similar to *Ec*IadA (Elias and Gygi, 2007) and *Cp*IadA (Shilov et al., 2007), size-exclusion chromatography (Figure 2B) indicated that *Fi*BAP is an octamer in solution (S1–S8 in Figure 4C), usually referred to as a “tetramer of dimers” (Figures 4C,D), as supported by symmetry-related molecule analysis. The initial dimer is formed when the β-sandwich domain of each monomer (Figure 4A) interacts with an adjacent monomer, mainly through hydrophobic interactions involving several residues (I2, I4, V34, V35, V38, L39, F41, and I46) in the loop between β4 and β5 (upper red box in Figure 4D). Additionally, the substrate-binding cavity of the catalytic domain (Figure 4A) participates in dimerization by providing the second point of contact between adjacent partners through salt bridges. These four salt bridges are formed by E107 (α2), K114 (α2), R143 (α3), and D149 (α3) of each monomer of the dimer (lower red box in Figure 4D). Therefore, the hydrophobic interactions between β-sandwich domains, followed by hydrogen (H)-bonds and salt bridges between catalytic domains of adjacent subunits results in dimer formation (Figure 4B and Supplementary Table 3). Remarkably, the corresponding region in structural homologs such as *Ec*IadA and *Cp*IadA does not appear to contain some of these salt bridges because the distances between charged amino acids are >4 Å in all cases except E114-K150 of *Ec*IadA (Supplementary Figure 3B) (Thoden et al., 2003; Park et al., 2017). Hence, we propose that the strong salt bridges may contribute to the thermostability of *Fi*BAP, as reported previously for a hyperthermophilic PIMT, in which deletion of the interfacial residues that interact through salt bridges affected the overall thermostability of the enzyme (Tanaka et al., 2004).

Subsequently, each dimer interacts with two adjacent dimers at three contact points (boxed in Figure 4E) to form a biologically active octamer that includes two contact points (S2–S4 interface and S1–S3 interface in Figure 4C) between similar structural elements α4, α5 of one dimer, and loops T213–L220, G95–L106, and I159–L169 of the adjacent dimer. We identified additional salt bridges formed at this interface between residues R174 (α4), D178 (α4), and E214 (α5) with E171, R131, and R201, respectively (pink and purple boxes in Figure 4E). However, these corresponding residues are conserved and form salt bridges in both *Ec*IadA and *Cp*IadA, revealing their significance in the formation of a stable quaternary structure. In addition, the third contact point (S2–S3 interface) at the dimer-dimer interface is mainly comprised of H-bonds formed between interfacial residues (brown box in Figure 4E). These interactions at different contact points are required in BAPs for their overall stability and to maintain their high oligomeric state.

**Figure 5:**
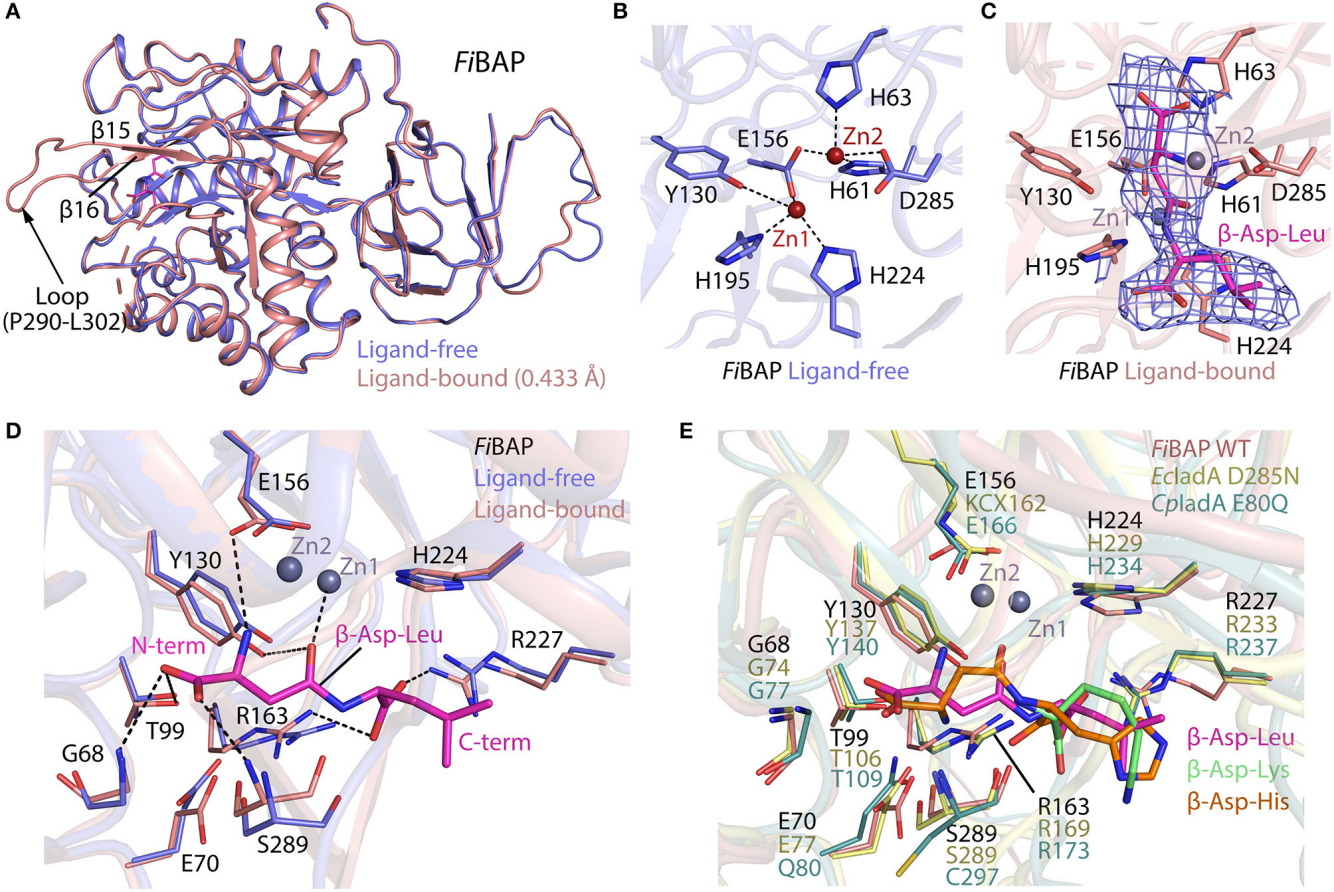
Comparison of ligand-bound and ligand-free structures of *Fi*BAP. **(A)** Alignment of ligand-bound and ligand-free *Fi*BAP monomers. Note that the P290–L302 loop is stabilized in the ligand-bound structure but not in the ligand-free model. **(B)** The active site of ligand-free *Fi*BAP containing binuclear zinc metal ions. Residues coordinating the metals are represented as sticks and H-bonds are shown as dotted lines. **(C)** The active site of ligand-bound *Fi*BAP. The 2Fo-Fc electron density maps of Zn^2+^ ions and β-DL are contoured at 1σ and colored blue. Residues of *Fi*BAP interacting with metal ions and β-DL are depicted as sticks. Zn^2+^ ions are represented as purple spheres and the bound β-DL is shown as magenta sticks. Ligands and side chains are colored by atom type (oxygen in red, nitrogen in blue, phosphorous in orange). **(D)** Comparison of residues coordinating the ligand between ligand-free and ligand bound models. Interactions between ligand-bound *Fi*BAP residues and atoms of the ligand are shown as dotted lines. **(E)** Comparison of the ligand-binding residues of *Fi*BAP bound to β-DL with their counterparts in ligand-bound homologs *Ec*IadA (PDB_1YBQ) and *Cp*IadA (PDB_5XGX). Conformational changes in side-chains are indicated. Residues coordinating the ligand are highly conserved among BAPs.

The ligand-free model included most amino acids except for residues D254–R259 and P290–L302, corresponding to the flexible loops in the molecule (Figure 4A). Intriguingly, the ligand-bound model included obvious electron density corresponding to loop P290–L302 between β14 and β15, which might have been stabilized upon ligand binding (Figure 5A). By contrast, loop D254–R259 of the Cbz-β-DL-bound *Fi*BAP structure remained disordered. Remarkably, the Cbz-β-DL-bound *Fi*BAP structure aligned with the ligand-free model with an RMSD of 0.433 Å, despite the good superimposition of the structural elements (Figure 5A). Such an unusually high RMSD value might be ascribed to the transformation of the missing loop (P290–L302) in the ligand-free structure into two stable antiparallel β-sheets (β14 and β15) in the ligand-bound structure (Figure 5A). Since this stabilized loop is located next to the substrate-binding site, it might function as a gate for substrate entry and/or product release (Park et al., 2017). Intriguingly, the crystal structure of *Fi*BAP bound to Cbz-β-DL included distinct electron density only for the β-DL moiety lacking carbamazepine (Cbz) in the active site (Figure 5C).

### The Active Site of *Fi*BAP

The substrate-binding site of BAPs, referred to as the “binuclear zinc center,” is located beneath the (β/α)8 TIM-barrel motif of the catalytic domain, and comprises two Zn^2+^ (Zn1 and Zn2) ions 3.2 Å apart from each other (dotted circle in Figure 4A). Zn1 is coordinated by E156, H195, and H224 residues, while Zn2 is bound by H61, H63, E156, and D285 (Figure 5B). In the metal coordination network, Zn1 interacts with Zn2 *via* E156 (Figure 5B). By contrast, a carbamylated lysine residue (KCX162) in Type-II *Ec*IadA binds two positively charged Zn ions, similar to E156 in Type-I *Cp*IadA (Figure 5E) (Thoden et al., 2003; Park et al., 2017). In addition, E156 in *Fi*BAP forms *a cis*-peptide bond with G155 in the loop between α4 and β10, which is conserved in the corresponding loop of *Cp*IadA between G165 and E166. The *cis*-peptide bond favors loop bending in *Fi*BAP, which positions E156 toward the metal, as opposed to the carbamylated lysine (KCX162) in *Ec*IadA, suggesting that *Fi*BAP belongs to the Type-I IadAs.

There is no significant perturbation in the coordination of the binuclear zinc center in the Cbz-β-DL-bound *Fi*BAP structure (Figure 5C). However, structural comparison of the residues around the ligand-binding site reveals subtle changes of ~1 Å in the side-chain conformations of some residues (G68, E70, Y130, R163, R227, and S289) between Cbz-β-DL-free and -bound forms (Figure 5D). Residues such as Y130, G68, and S289 are placed closer to the ligand than those in the ligand-free structure, thereby assisting coordination and stabilization, while R227 and R163 move away to provide space for ligand binding. Notably, the role of the conserved Y130, which interacts with the O06 atom of the substrate, has been studied, and the Y137F mutation in *Ec*IadA (corresponding to Y130 in *Fi*BAP) reduced the rate of catalysis by three orders of magnitude (Marti-Arbona et al., 2005b). Accordingly, the conserved Y130 in *Fi*BAP not only contributes to stabilization of the bound substrate, but also acts as a Lewis acid with the phenolic hydroxyl group of its side chain during hydrolysis of the peptide bond adjacent to Asp of the dipeptide (Marti-Arbona et al., 2005a; Park et al., 2017). The other surrounding residues that help to stabilize the bound Cbz-β-DL are the amino groups of main-chain residues S289 and G68, while T99 interacts with O04 and O05 atoms of the side-chain of the Asp residue of β-aspartyl leucine to stabilize the N-terminus of the dipeptide during substrate recognition. Additionally, R163 and R227 interact with O01 and O03 atoms and stabilize the C-terminal end of the peptide. When the substrate is recognized and stabilized, other residues such as E156, Y130, and E70 partake in acid-base catalysis during cleavage of the peptide bond via zinc metal ions Zn1 and Zn2 (Figure 5D). As stated above, all residues involved in the recognition and catalysis of a substrate are highly conserved among homologs (Figure 5E). Intriguingly, the ligand-bound structures of *Ec*IadA and *Cp*IadA were only obtained following mutation of conserved residues involved in metal coordination and catalysis (D285N and E80Q, respectively). By contrast, the structure of Cbz-β-DL-bound *Fi*BAP was determined directly from wild-type *Fi*BAP. This observation may indicate that the crystallization conditions (temperature <20°C and pH < pH 5) could have inactivated the thermostable *Fi*BAP enzyme, favoring formation of the substrate (β-DL)-bound complex structure without being cleaved, as seen in the electron density map (Figure 5C).

### Complementation of a Leucine Auxotroph by *Fi*BAP

**Figure 6:**
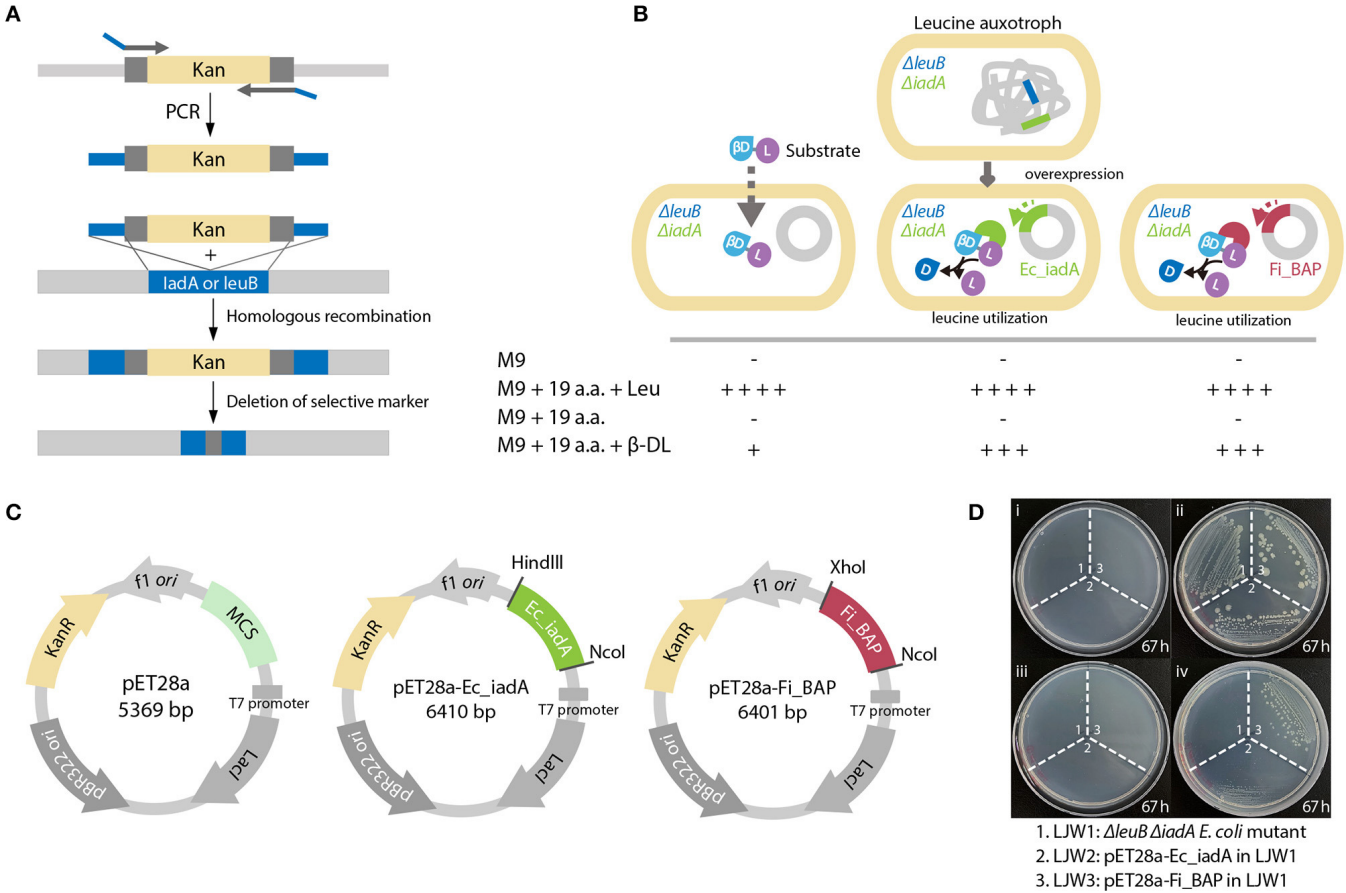
*Fi*BAP complementation assay of the Leu auxotrophic *leuB iadA E. coli* double mutant grown on isoAsp peptides. **(A)** Strategy for obtaining the Leu auxotrophic Δ*leuB* Δ*iadA E. coli* double mutant using the RED/ET recombination method. **(B)** Comparison of growth phenotypes for three *E. coli* mutant strains (LJW1, the Δ*leuB* Δ*iadA* double mutant; LJW2, the LJW1 strain harboring the pET28a-IadA overexpression plasmid; and LJW3, the LJW1 strain harboring the pET28a-*Fi*BAP overexpression plasmid) grown in four different types of medium: (i) M9 minimal medium (M9); (ii) M9 minimal medium containing 20 amino acids (M9-20aa); (iii) M9 minimal medium containing 19 amino acids (Leu^−^, M9-19aa); and (iv) M9 minimal medium containing 19 amino acids (Leu^−^) and 1.6 mM β-Asp-Leu (M9_19aa_β-DL). Expected growth phenotypes are marked as + or –. **(C)** Schematic representation of plasmid construction for recombinant proteins. Genes encoding *Ec*IadA and *Fi*BAP were ligated into the pET28a expression vector digested with appropriate restriction enzymes. **(D)** Photographs of three *E. coli* mutant strains on various M9 plates after a 67 h incubation at 37°C.

To validate whether *Fi*BAP can cleave β-DL *in vivo*, we constructed a Leu auxotrophic *E. coli* BL21 (DE3) strain by deleting the *leuB* gene encoding 3-isopropylmalate dehydrogenase and the *iadA* gene encoding BAP in *E. coli* (Figure 6A). We presumed that the hydrolysis of β-DL by *Fi*BAP could provide Leu as a nutrient for the Leu auxotrophic *E. coli* mutant, thereby supporting its growth on M9 minimal medium lacking Leu (Figure 6B). As expected, the Δ*leuB* and Δ*iadA* double mutant grew in M9 medium supplemented with 20 amino acids, but did not grow in M9 medium containing 19 amino acids without Leu (Leu^−^; Figure 6D). Next, to investigate the effect of overexpression of *Fi*BAP and *EcIadA* on bacterial growth, we constructed the pET28a-Ec_iadA and pET28a-Fi_BAP vectors (Figure 6C) and heterologously expressed the *FiBAP* and *iadA* genes in Δ*leuB* and Δ*iadA* double mutants of *E. coli* BL21 (DE3), respectively. The Δ*leuB* Δ*iadA* mutant did not grow on M9 medium supplemented with 19 amino acids (Leu^−^) in the presence of β-DL during 3 days of incubation, whereas the Δ*leuB* Δ*iadA* double mutant harboring the pET-*iadA* plasmid grew on M9 medium with 19 amino acids (Leu^−^) supplemented with β-DL, as described previously (Marti-Arbona et al., 2005a). Remarkably, the double mutant containing the pET-*Fi*BAP plasmid could also grow on M9 medium with 19 amino acids (Leu^−^) in the presence of β-DL even at a suboptimal temperature for *Fi*BAP (Figure 6D). This result strongly indicates that *Fi*BAP can also cleave the β-DL dipeptide to facilitate Leu utilization for bacterial growth, suggesting that *Fi*BAP activity enables *E. coli* to utilize β-aspartyl peptides as nutrient sources for cell growth.

**Figure 7:**
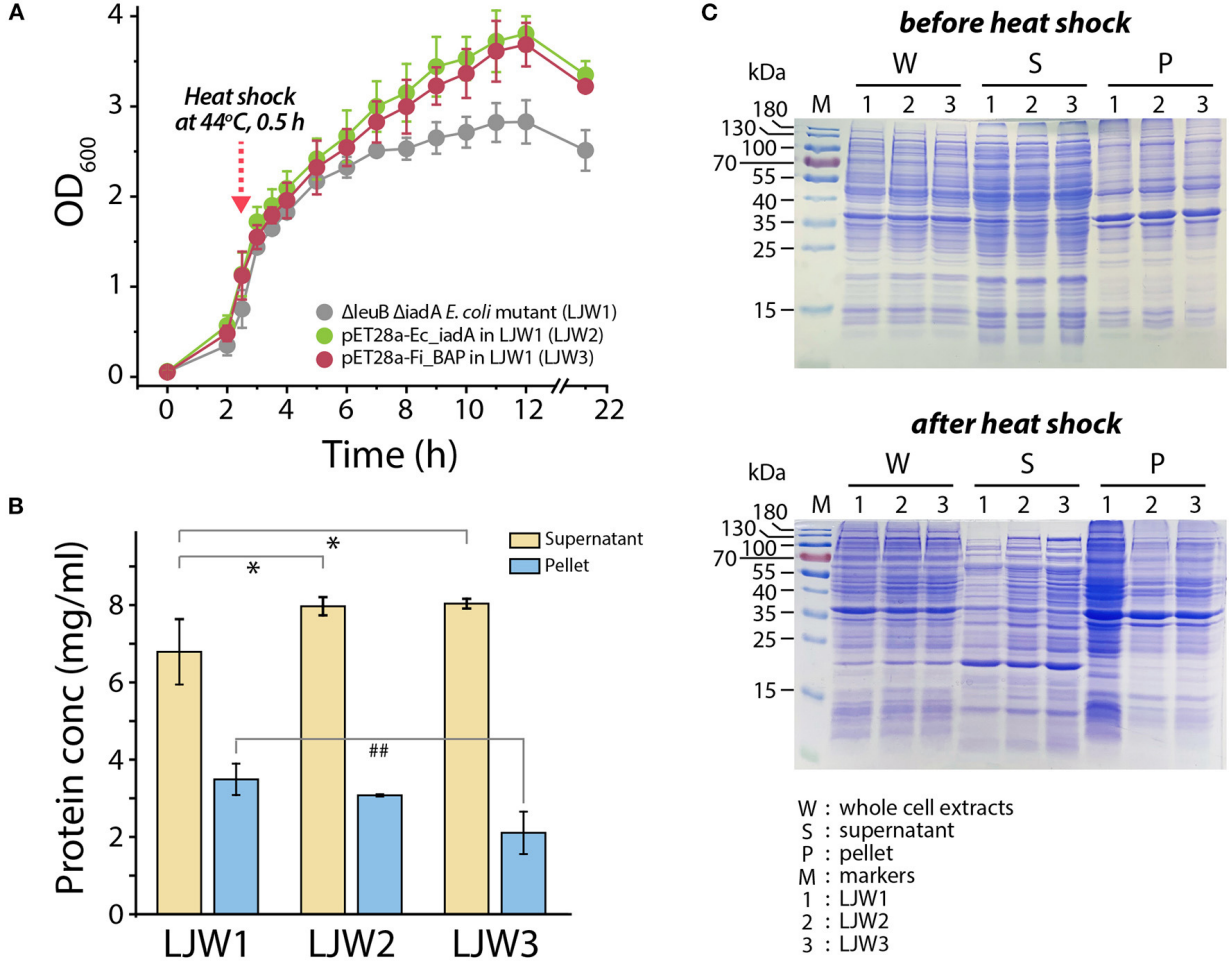
Bacterial growth and protein pattern analysis after heat treatment. **(A)** Growth curve of *E. coli* mutant strains (LJW1, LJW2, and LJW3) in LB media at 37°C. Heat shock treatment was performed at 44°C for 30 min when cells reached the mid-exponential phase (OD_600_ of 1.0–1.5). **(B)** Quantification of soluble (supernatant) and insoluble (pellet) protein in heat-treated cells. Group differences were assessed using one-way analysis of variance (ANOVA), followed by LSD test (^*^p < 0.05 vs. WT supernatant and ^##^*p* < 0.01 vs. WT pellet). **(C)** Comparison of whole protein, soluble (supernatant) protein, and insoluble (pellet) protein in heat-treated and non-heat-treated cells by SDS-PAGE analysis.

Accordingly, we hypothesized that heat stress may cause accumulation of misfolded and/or aggregated polypeptides, including the formation of isoAsp residues, resulting in cellular growth retardation. If so, then overexpression of *Fi*BAP may alleviate the bacterial growth defect. To validate this, we monitored the growth profiles of the Δ*leuB* Δ*iadA* double mutant grown on LB medium with and without *Fi*BAP upon heat shock at 44°C. Consequently, after a 30 min heat shock treatment, the growth rate and cellular production of the Δ*leuB* Δ*iadA* mutant were decreased by 30% (based on the OD_600_ value) compared with those of mutants expressing *Fi*BAP and *Ec*IadA, respectively (Figure 7A). In addition, when expressing BAP, the Δ*leuB* Δ*iadA* mutant exhibited a 15–40% decrease in the total protein concentrations of pellets, whereas the concentrations of soluble cytosolic proteins were increased by 20% (Figure 7B). Furthermore, differential protein pattern analysis by SDS-PAGE clearly indicated that expression of *Fi*BAP could affect the soluble cytosolic protein profiles of the mutant *E. coli* strains, suggesting that abnormal proteins including isoAsp residues could be efficiently degraded by BAP activity under heat stress conditions (Figure 7C).

**Figure 8:**
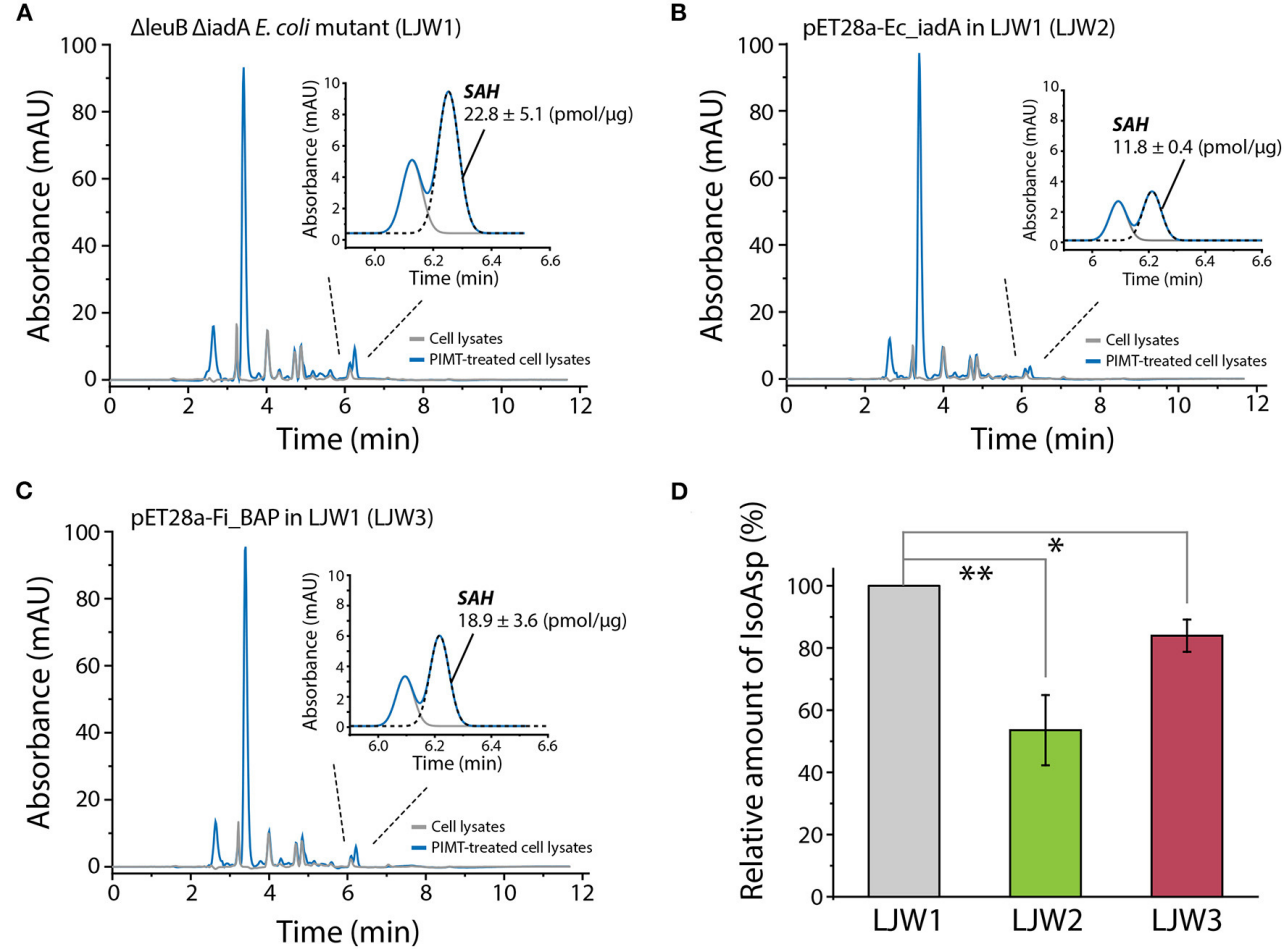
RP-HPLC analysis of SAH for quantification of isoAsp residues in heat-shocked LJW1, LJW2, and LJW3 strains. **(A–C)** RP-HPLC analysis of SAH in LJW1, LJW2, and LJW3 strain and quantification of isoAsp by calculating the SAH peak area. **(D)** Relative amount of isoAsp in each heat- treated cell. Group differences were assessed using one-way analysis of variance (ANOVA), followed by LSD test [^**^p < 0.01 and ^*^p < 0.05 vs. WT (LJW1)].

Finally, to investigate whether overexpression of *Fi*BAP and *Ec*IadA can reduce the isoAsp-containing protein aggregates within cells, we determined the concentration of S-adenosyl homocysteine (SAH) as the PIMT-mediated by-product derived from the isoAsp-containing peptides in heat-treated mutant strains. Indeed, RP-HPLC analysis of cell lysates demonstrated that the concentrations of SAH in both mutants expressing *Fi*BAP and *Ec*IadA were lower by 20 and 50%, respectively, than that of the Δ*leuB* Δ*iadA E. coli* mutant (LJW1) negative control (Figures 8A–C). These results indicate that the relative amount of isoAsp-containing proteins was decreased significantly in mutants overexpressing *Ec*IadA (LJW2) or *Fi*BAP (LJW3) compared with WT (LJW1) (Figure 8D), suggesting that BAP can hydrolyze isoAsp peptide bonds in the protein aggregates, and thereby alleviate the bacterial growth defect under heat shock conditions.

## Discussion

Protein folding and misfolding are critical for correct cellular function and regulation. Indeed, compelling evidence suggests that misfolded and damaged protein aggregates contribute to cell dysfunction and tissue damage, leading to various diseases (Dobson, 2003; Goldberg, 2003). For example, misfolding of amyloidogenic proteins is strongly associated with the pathogenicity of neurodegenerative diseases such as Alzheimer's, Parkinson's, and Huntington's diseases, and AA amyloidosis. Even metabolic syndromes such as type 2 diabetes can be triggered by misfolded protein aggregates (Moreno-Gonzalez and Soto, 2011). It has been reported that up to 30% of newly synthesized proteins may be misfolded and/or aggregated due to errors in translation or post-translational modifications, including the formation of abnormal amino acid residues (Schubert et al., 2000). In particular, unwanted protein aggregates resulting from the formation of isoAsp residues are an example of spontaneous age-related protein damage in cells, and their degradation and/or removal via deamination and dehydration is essential to prevent cellular cytotoxicity and aging (Ogé et al., 2008). To ensure cellular viability in response to starvation, organisms also tend to preserve protein homeostasis through protein repair systems using several strategies such as refolding, degrading, or sequestrating misfolded polypeptides (Goldberg, 1972; Finn and Dice, 2006; Chen et al., 2011).

In extremophiles thriving under harsh environments, in which proteins are vulnerable to protein inactivation and aggregation, cellular protein repair systems play a pivotal role in protein quality control (PQC) to support cellular integrity and survival (De Castro et al., 2006; Chen et al., 2011; Coker, 2019). In the present study, we functionally characterized the hyperthermophilic M38 β-aspartyl peptidase *Fi*BAP, a protein repair enzyme involved in hydrolysis of isoAsp residue-containing peptides in *F. islandicum* AW-1. Since the extremely thermophilic *F. islandicum* AW-1 belongs to the order Thermotogales, an ancient branch of the bacterial kingdom, this bacterium possesses primordial characteristics, making it an excellent model system for investigating how extremophiles have evolved mechanisms to prevent protein damage and/or denaturation under harsh environments (Singleton and Amelunxen, 1973). Although several functional studies on BAPs from mesophilic organisms have been reported, including human (Ryttersgaard et al., 2002), *E. coli* (Marti-Arbona et al., 2005b), and *Colwellia psychrerythraea* (Marti-Arbona et al., 2005b; Park et al., 2017), hyperthermophilic homologs remain uncharacterized.

Phylogenetic analysis based on the primary sequences of its homologs revealed that *Fi*BAP is distantly related to Type II IadA enzymes, suggesting that *Fi*BAP belongs to an ancient form of the BAP family in microorganisms (Figure 1 and Supplementary Figure 1). Our detailed characterization revealed that *Fi*BAP is a hyperthermophilic BAP with a T_m_ value of 96.8°C in the presence of Zn^2+^ that forms a tetramer of dimers (Figure 2), consistent with the first crystal structures of octameric Cbz-β-DL-free and -bound BAPs, and size-exclusion chromatography and symmetry-related molecule analysis (Figures 2B, 4C). The octameric state of *Fi*BAP was compared with the quaternary structures of structural homologs of *Ec*IadA and *Cp*IadA (Supplementary Figure 3A), indicating no significant difference in their overall architecture (Supplementary Figure 3A). Although the crystal structure of *Fi*BAP superimposed well with those of mesophilic *Ec*IadA and *Cp*IadA (Figure 4B), its mesophilic counterparts do not appear to include several of the strong salt bridges (Supplementary Figure 3B). Remarkably, the catalytic domain of *Fi*BAP participates in dimerization through salt-bridges via R143, K114, E107, and D149 from each monomer, and this is largely responsible for the high thermostability (Figure 4). This observation is consistent with the study of a hyperthermophilic PIMT, in which mutation of the interfacial residues (D204 and D205) to alanine significantly affected thermostability (Gary and Clarke, 1995). Regarding substrate recognition and ligand stabilization, residues such as G68, T99, Y130, and S289 are highly conserved among all homologs (Figure 5E). Notably, residues T99 and S289 appear to be involved in recognition of the N-terminal iso-Asp side chain of the substrate, similar to the roles played by T57 and S59 in protein isoAsp methyltransferase (PIMT) that recognizes abnormal proteins containing isoAsp residues during protein repair (Chen et al., 2011). Consistently, the conserved Y130 residue, which serves the dual purpose of acting as a Lewis acid and supporting ligand stabilization, is also present in PIMT (Y55). Although, the amino acids mediating isoAsp recognition appear to be conserved with PIMT, the role of the binuclear zinc center in BAPs in cleaving the peptide bond is not conserved in PIMT. Taken together, the results indicate that *Fi*BAP is a thermostable alternative protein repair enzyme that possesses conserved residues for isoAsp residue recognition and for efficient Zn^2+^-mediated peptide bond hydrolysis.

In contrast to the crystal structures of BAP homologs inactivated by mutations (Thoden et al., 2003; Park et al., 2017), the crystal structure of the ligand-bound form of *Fi*BAP was obtained without any significant cleavage of bound β-DL (Figures 5A,C,E). This could be explained by various reasons. Firstly, crystallization was carried out at below room temperature (20°C) in a low-pH buffer (pH < 5.0), which could have affected the enzyme activity (Figures 2C,D). Secondly, we synthesized Cbz-β-DL dipeptide as a substrate analog inhibitor in which benzyl chloroformate (Cbz) is a carbobenzyloxy protecting group that protects the substrate amino group. Previous studies have shown clearly that Cbz acts as an inhibitor of several enzymes systems including human alpha-thrombin (De Simone et al., 1997) and sortase cysteine transpeptidase (Jacobitz et al., 2014). Consistently, our substrate analog (Cbz-β-DL) containing Cbz inhibited enzyme activity in our enzyme assays (data not shown), which suggests that wt *Fi*BAP did not cleave the Cbz-β-DL dipeptide. Therefore, these modifications worked in our favor to obtain the crystal structure of the wild-type enzyme complexed with its substrate. However, the fate of Cbz in the substrate-bound enzyme is ambiguous due to the absence of electron density for the Cbz moiety in the structure. Interestingly, the location of an uncleaved β-DL based on the electron density map (Figure 5C) coincides precisely with the dipeptide (β-Asp-Lys or His) location described in previous studies (Thoden et al., 2003; Marti-Arbona et al., 2005a; Park et al., 2017) (Figure 5E). Thus, it seems probable that the Cbz entity may be cleaved by hydrogenolysis before the N-terminal end of the dipeptide enters the substrate-binding cavity, since the benzyl ring of Cbz (if uncleaved) might cause steric hindrance within the cavity. Furthermore, the second amino acid following Asp in the dipeptide (Leu in β-DL) is unlikely to interact with any surrounding residues of *Fi*BAP (Figures 5D,E).

The primordial *Fi*BAP is a unique metallopeptidase that is presumably involved in the sequestration of unwanted forms of peptides. Notably, *Fi*BAP is the most abundant and highly expressed protein when *F. islandicum* AW-1 is grown on feather keratins under starvation conditions (Kang et al., 2020). Although this enzyme is not one of the major endo-type membrane proteases, but rather a protease involved in keratin degradation, its physiological role seems to be important for cellular survival due to the formation of isopeptides that contribute to thermostability, as observed in other anaerobic pathogens (Liu et al., 2016), as well as a providing a source of amino acids for new protein synthesis (Prouty and Goldberg, 1972). The formation of isoAsp residues has received much attention because it is a major structural modification that contributes to the inactivation, aggregation, and malfunction of proteins (Aswad, 1995; Cournoyer et al., 2005). Spontaneous isopeptide bond formation is accelerated at elevated temperatures, and intermolecular amide bonds help proteins to be more thermostable (Zakeri and Howarth, 2010; Si et al., 2016). However, such chemical modifications are not always favorable for cellular viability due to the accumulation of potentially harmful isoAsp peptides in the brain and other tissues (Cantor et al., 2009). Furthermore, balance between the formation and decomposition of isopeptide bonds via isoAsp peptidase is crucial to bacterial survival. Indeed, the growth profiles of the Δ*leuB* Δ*iadA* double mutant of *E. coli* BL21 clearly indicated that heat shock for 30 min significantly retarded bacterial growth, presumably due to an increase in the accumulation of abnormal proteins (Figure 7A). Notably, expression of *Fi*BAP, even at suboptimal temperatures ranging from 37 to 44°C, may be sufficient to support bacterial growth through efficient degradation of isoAsp residue-containing proteins. Although the amount of IsoAsp in mutant expressing *Fi*BAP was not decreased as much as in mutant expressing *Ec*IadA, levels of alleviation of growth defect were similar. This result suggests that enzymatic activity of *Fi*BAP is not limited toward isoAsp containing peptides but also includes a broad range of protein aggregates which is related to the interpretation of our MS data (Figures 3, 7B,C, 8).

Unlike BAPs, which are mainly present in extremophiles and several enteric bacteria, PIMT is a conserved and nearly ubiquitous enzyme present in all forms of life that catalyzes the transfer of the methyl group from AdoMet to α-carboxyl of an isoAsp site, resulting in the rapid decomposition of methyl esters to succinimide intermediates (Skinner et al., 2000). Therefore, like isoAsp peptidase, PIMT is a useful enzyme for repairing isoAsp damage in all organisms. Nevertheless, when decomposition of isopeptide bonds is a prerequisite for balancing nutrient availability and thermostability under extreme environments, PIMT is unlikely to be an efficient catalyst in terms of the rate of isopeptide decomposition and formation. Indeed, the formation of succinimide by PIMT resulting from isoAsp-containing peptides generates a mixture of isoAsp-Xaa and Asp-Xaa with a stoichiometric ratio of 7:3 (Aswad et al., 2000). However, BAP can directly hydrolyze isopeptide bonds via the β-carboxylate group of isoAsp, resulting in the release of Asp (Marti-Arbona et al., 2005a), and this might be a more efficient repair mechanism for handing β-aspartyl-containing peptides. Furthermore, proteolytic pathways function during starvation to liberate amino acids, and this may be beneficial for dietary protein digestion and cellular protein turnover (Finn and Dice, 2006).

Regarding the functional and physiological roles of *Fi*BAP, we offer two suggestions explaining why this enzyme is conserved in all extremely thermophilic anaerobes, and why it is so abundant in *F. islandicum* AW-1 during keratin degradation. Firstly, it is one of the key enzymes involved in maintaining proteostasis in thermophiles thriving in harsh environments. By directly hydrolyzing peptide bonds in isoAsp-containing peptides, it can efficiently control aggregated, modified, or damaged proteins, thereby supporting the viability of thermophiles at elevated temperatures. Secondly, under starvation conditions, when insoluble keratins are the only nutrient available for cellular growth, this enzyme may utilize β-aspartyl peptides as a source of amino acids for protein biosynthesis. This hypothesis is supported by previous studies showing that *Fi*BAP is expressed at high levels under starvation conditions (Kang et al., 2020), suggesting that nutrient depletion acts as a stress condition that stimulates *Fi*BAP expression and enzyme activity. Furthermore, peptidase-deficient *E. coli* leucine auxotrophic strains did not grow in M9 minimal media containing 19 amino acids (Leu^−^) and β-DL. However, expression of *Ec*IadA or *Fi*BAP enabled the *E. coli* mutant to grow in this medium, implying that *Fi*BAP contribute to increasing the availability of nutrients (Viola, 2001), thereby assisting the maintenance of cellular growth under stressful conditions (Figure 6). Thus, *Fi*BAP may not be a membrane bound endo- or exo-type keratinolytic protease (Kang et al., 2020), but it appears to be strongly associated with the degradation of abnormal proteins as well as intracellular keratin peptides to provide a source of amino acids for protein biosynthesis under stress conditions.

## Data Availability Statement

The original contributions presented in the study are included in the article/Supplementary Material, further inquiries can be directed to the corresponding author/s.

## Author Contributions

JL, ID, HJ, SL, and D-WL formulated the research plan, carried out experiments, analyzed and interpreted the data, and drafted the manuscript. JL, ID, SL, and D-WL participated in the design of the study and analyzed and interpreted the data. SL and D-WL conceived, planned, and supervised the study. All authors contributed to the article and approved the submitted version.

## Conflict of Interest

The authors declare that the research was conducted in the absence of any commercial or financial relationships that could be construed as a potential conflict of interest.
