# Supplementary material for: Functional Characterization of Primordial Protein Repair Enzyme M38 Metallo-Peptidase From Fervidobacterium islandicum AW-1
Source: Front Mol Biosci. 2020 Dec 17;7:600634. doi: 10.3389/fmolb.2020.600634 (PMC7774594; doi:10.3389/fmolb.2020.600634)
Supplement: Supplementary file 1 [file Data_Sheet_1.docx]

# **Supplementary files**

# **Functional characterization of primordial protein repair enzyme M38 β-aspartyl peptidase from *Fervidobacterium islandicum* AW-1**

Jae Won La^1,¶^, Immanuel Dhanasingh2,¶, Hyeonha Jang^3^, Sung Haeng Lee^2^, and Dong-Woo Lee^1,^*

^1^Department of Biotechnology, Yonsei University, Seoul 03722, South Korea.

^2^Department of Cellular and Molecular Medicine, Chosun University School of Medicine, Gwangju 501-759, South Korea.

^3^School of Applied Biosciences, Kyungpook National University, Daegu 41566, South Korea.

Running Title: *Fervidobacterium islandicum* M38 β-aspartyl peptidase

^*^To whom correspondence should be addressed:

**Dong-Woo Lee,** Department of Biotechnology, Yonsei University, Seoul 03722, South Korea.

Tel.: +82-2-2123-2886; Fax: +82-2-362-7265; E-mail: [leehicam@yonsei.ac.kr](mailto:leehicam@yonsei.ac.kr)

^,¶^These authors contributed equally to this work.

**Number of figures and tables = 8 figures and 1 table**

**Number of supplementary figures and tables = 3 figures and 3 tables**

**Table S1. Purification** **of *Fi*BAP**

| **Fraction of purification** | **Volume**  **(ml)** | **Protein conc**  **(mg/ml)** | **Total protein (mg)** | **Total activity**  **(U)** | **Specific activity**  **(U/mg)** | **Yield**  **(%)** | **Fold purification** |
| --- | --- | --- | --- | --- | --- | --- | --- |
| Whole cell extract | 40 | 12.75 | 510 | 3672 | 6.83 | 100 | 1.00 |
| Heat-treated  cell extract | 35 | 5.80 | 203 | 1693 | 10.11 | 46.1 | 1.48 |
| Affinity chromatography | 36 | 1.43 | 51.5 | 664 | 17.32 | 18.1 | 2.54 |
| Gel filtration chromatography | 4 | 0.41 | 1.64 | 48 | 27.68 | 1.31 | 4.05 |

**Table S2. List of recombinant feather keratin hydrolysates**

| **Peptide sequence (N-terminal)** | **Observed m/z** | **Z** | **m/z error (Da)** | **Frequency** | **Peptideshaker conf** | **Origin** |
| --- | --- | --- | --- | --- | --- | --- |
| SSGGFGFGGLGCFNGWRARYPC | 805.30 | 3 | 0.94 | 2 | 100 | Chr2_FK4 |
| SGRLTPPGTLHPTAMS | 547.03 | 3 | 0.09 | 4 | 100 | Chr2_FK4 |
| ASPVPRILTPF | 599.35 | 2 | 0.90 | 8 | 57 | Chr2_FK4 |
| HSSIKASPVPRILTP | 536.11 | 3 | 0.79 | 19 | 50 | Chr2_FK4 |
| FDLSGFGSRYCGRRCPPC | 1098.16 | 2 | 0.15 | 3 | 83 | Ch27_FK12 |
| SEGVPITSGGFDLSGFGSRYCGRRCPPC | 1027.33 | 3 | 0.87 | 5 | 71 | Chr27_FK12 |
| VVGSSTSAAVGSILSSEGVPITSGGFD | 828.00 | 3 | 0.08 | 4 | 71 | Chr27_FK12 |

**Table S3. Interfacial interactions between subunits S1 and S2.**

| **Hydrogen bonds** | | | |
| --- | --- | --- | --- |
| **##** | [**Subunit**](javascript:openWindow('pi_ipage_atom1.html',400,250);) **S1** | [**Distance (Å)**](javascript:openWindow('pi_ipage_atmdist.html',400,250);) | **Subunit S2** |
| 1 | Y24[OH] | 3.78 | V27[O] |
| 2 | R143[NH2] | 2.89 | E107[OE2] |
| 3 | R143[NH2] | 3.09 | F137[O] |
| 4 | Y110[OH] | 3.31 | L147[O] |
| 5 | K114[NZ] | 2.68 | D149[OD1] |
| 6 | V27[O] | 3.78 | Y24[OH] |
| 7 | E107[OE2] | 2.89 | R143[NH2] |
| 8 | F137[O] | 3.09 | R143[NH2] |
| 9 | L147[O] | 3.31 | Y110[OH] |
| 10 | D149[OD1] | 2.68 | K114[NZ] |

| **Salt bridges** | | | |
| --- | --- | --- | --- |
| **##** | [**Subunit**](javascript:openWindow('pi_ipage_atom1.html',400,250);) **S1** | [**Distance (Å)**](javascript:openWindow('pi_ipage_atmdist.html',400,250);) | **Subunit S2** |
| 1 | R143[NH1] | 3.52 | E107[OE2] |
| 2 | R143[NH2] | 2.89 | E107[OE2] |
| 3 | K114[NZ] | 2.68 | D149[OD1] |
| 4 | K114[NZ] | 3.64 | D149[OD2] |
| 5 | E107[OE2] | 3.52 | R143[NH1] |
| 6 | E107[OE2] | 2.89 | R143[NH2] |
| 7 | D149[OD1] | 2.68 | K114[NZ] |
| 8 | D149[OD2] | 3.64 | K114[NZ] |

**
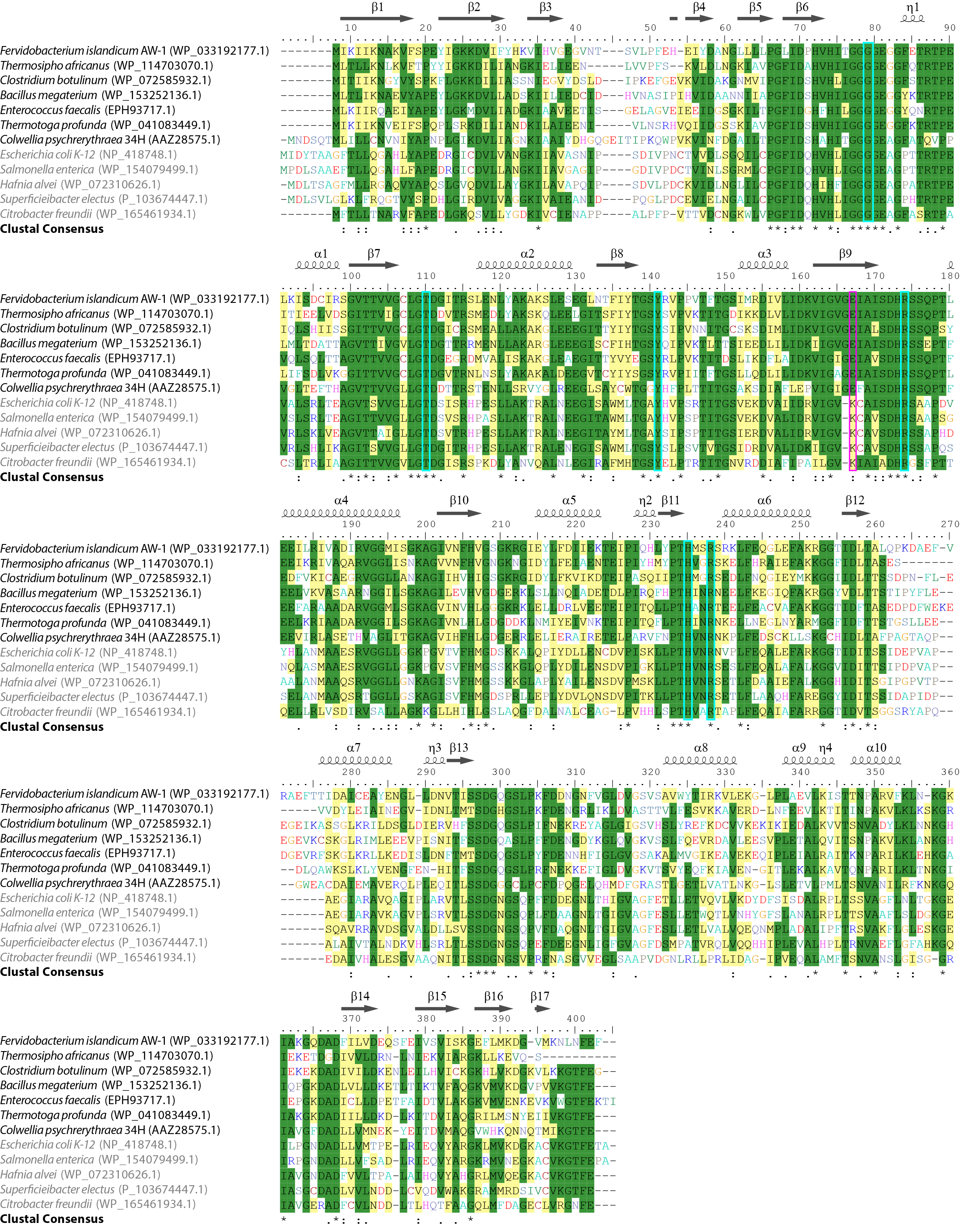
**

Figure S1. Alignment of FiBAP and its microbial homologs. Amino acid sequence alignment was generated by ClustalX (under BioEdit) of representative sequences of BAPs in different species. The residues of FiBAP involved in α-helices or β-strands are indicated by helical lines and arrows, respectively. Secondary structure prediction was carried out by using the ESPript 3.0 program. Type-I IadAs and Type-II IadAs were indicated in black and grey, respectively. NCBI protein database accession numbers for each individual protein sequence are indicated in parenthesis. Cyan box indicates conserved residue contributing to recognition and catalysis of a substrate, whereas red box indicates Glu as the metal binding residue in the active site of Type I BAP. Sequence similarities and secondary structure information from aligned sequences were expressed above.

**
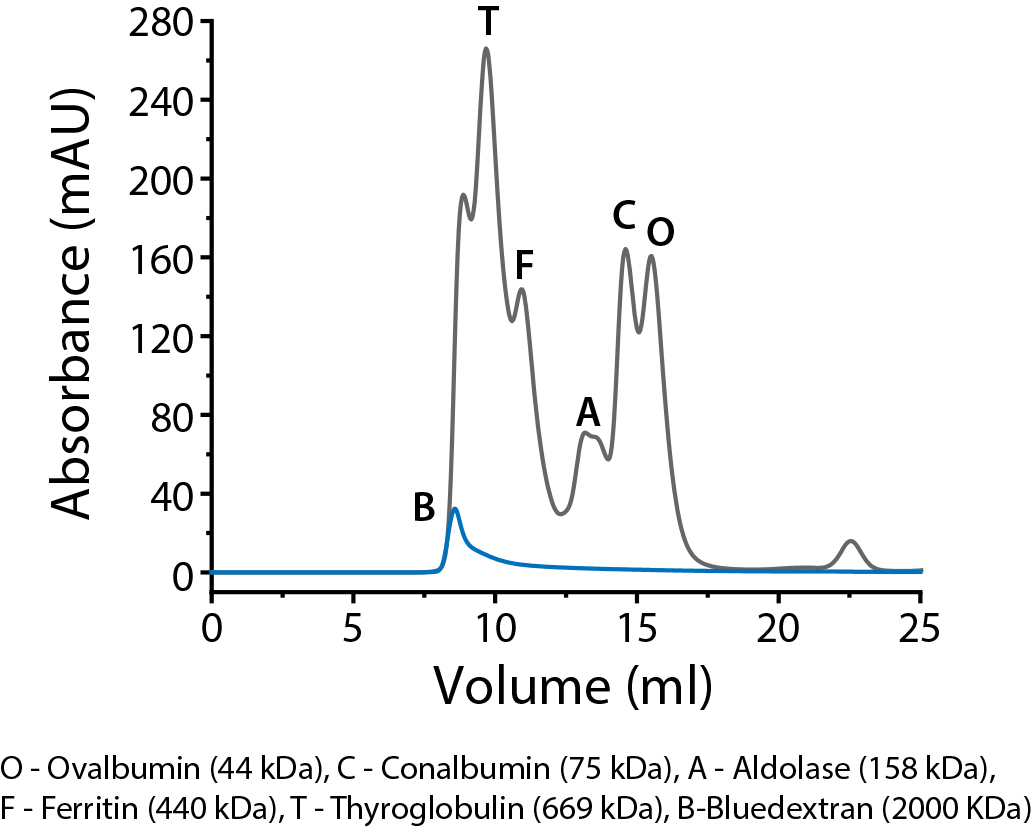
**

Figure S2. Elution profile of standard proteins. Gel filtration chromatography was performed using a Superdex 200 10/300 GL column (GE Healthcare, USA) equilibrated with 25 mM Tris-HCl buffer (containing 150 mM NaCl; pH 7.5), which was run at a flow rate of 0.2 ml/min, and the elution profile was monitored at 280 nm. The column was calibrated with blue dextran (2,000 kDa), thyroglobulin (669 kDa), ferritin (440 kDa), aldolase (158 kDa), conalbumin (75 kDa), and ovalbumin (44 kDa) as standards, and their elution positions are indicated at the top of each peak.

**
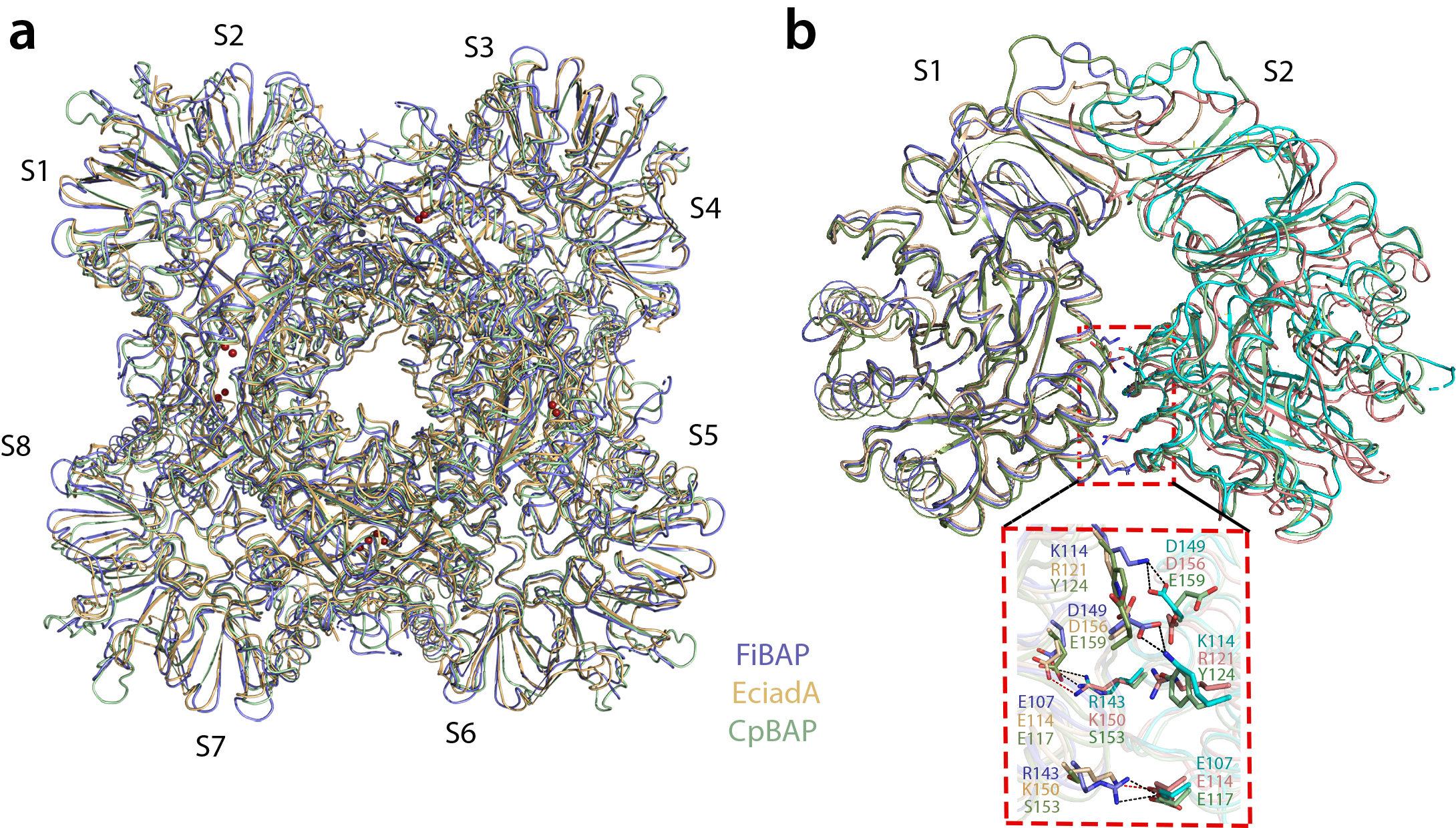
**

Figure S3. Overall structures of FiBAP and its mesophilic homologs. a) Comparison of the oligomeric states between FiBAP (Colored in slate blue) and homogs CpBAP (colored in smudge green) and EciadA (Colored by yellow orange). Each sub-unit (S1-S8) forms an octameric state. The Zinc metal is shown as brick colored in red. b) Comparison of the monomer-monomer interactions between FiBAP and its homologs. The view is similar to Fig. 4d (see the main text). c) The interactions between catalytic domains by the amino acids that form salt-bridge in FiBAP are zoomed in and compared with the side chain orientations of other two homologs.
